# Development of REC1: A Reverse Vaccinology-Derived Multi-Epitope Vaccine Candidate Against Brucellosis

**DOI:** 10.3390/vaccines14100891

**Published:** 2026-10-12

**Authors:** Shuang Liu, Yunpeng Dai, Fang Huang, Zixin Gao, Zeliang Chen, Miao Long, Shanhu Li

**Affiliations:** 1 Key Laboratory of Livestock Infectious Diseases, Ministry of Education, College of Animal Science and Veterinary Medicine, Shenyang Agricultural University, 120 Dongling Road, Shenyang 110866, Chinazeliangchen@yahoo.com (Z.C.); 2 National Key Laboratory of Advanced Biotechnology, Academy of Military Medical Sciences, Beijing 100071, China; 3 Department of Nuclear Medicine, China-Japan Union Hospital of Jilin University, Changchun 130033, China

**Keywords:** brucellosis, multi-epitope vaccine candidate, reverse vaccinology, immunoinformatics, epitope validation, 3D structure modeling, IFN-γ ELISpot

## Abstract

**Background/Objectives**: Brucellosis is a widespread intracellular zoonosis that continues to impose substantial public-health and economic burdens in endemic regions. No human brucellosis vaccine has yet been approved, and most existing multi-epitope candidates rely solely on computational predictions, without population-specific human leukocyte antigen (HLA) optimization or in vivo epitope validation. REC1 is therefore an early-stage, proof-of-concept, human-oriented multi-epitope candidate, with epitopes screened against high-frequency HLA alleles in the Chinese population. This study developed a multi-epitope vaccine candidate, REC1, using an integrated reverse-vaccinology workflow. **Methods**: We predicted Major Histocompatibility Complex (MHC) I and II epitopes against high-frequency HLA alleles representative of the Chinese population (AFND). Population coverage of the final incorporated epitope set was estimated with the IEDB Population Coverage tool under Hardy–Weinberg equilibrium, while paired HLA-DQ heterodimers, which the tool cannot model, were evaluated independently from Chinese DQA1-DQB1 haplotype frequencies. Epitopes supported by in vivo immunoreactivity were then assembled into the modular REC1 construct, which we characterized in silico for physicochemical properties, structural architecture, and simulated immune responses. **Results**: IFN-γ ELISpot shortlisted seven MHC-I and five MHC-II candidate immunoreactive epitopes derived from five *Brucella* antigens, of which five MHC-I and five MHC-II (one per antigen) were incorporated into REC1. The incorporated set was predicted to cover 91.17% of the China Oriental population on a conservative class I+II estimate (98.88% when HLA-DP was included; the HLA-DQ component alone covered 58–79%, 68.17% in Han). Epitope-level BLAST showed that all twelve shortlisted epitopes were 100% conserved across major *Brucella* species, except that the two Omp31 epitopes are absent from the Omp31-deleted *B. abortus*. The 35.86 kDa REC1 construct was predicted to be highly stable (instability index = 25.25) and hydrophilic, with a distinct architecture in which the carrier domain is ordered and the epitope region is disordered. Immune simulations suggested that REC1 can induce Th1-polarized humoral and cellular responses and may support long-term immunological memory. Molecular docking predicted that the L7/L12 carrier forms well-defined interfaces with TLR2, CD14, C1q, and DC-SIGN, and a representative peptide–MHC model confirmed canonical groove binding of a CTL epitope. **Conclusions**: REC1 exhibits favorable in silico properties, and its embedded peptides show IFN-γ immunogenicity in mice (candidate immunoreactive epitopes), lending preliminary support to its value as a multi-epitope vaccine candidate. HLA-matched validation and challenge studies are still needed to establish protective efficacy. Our study also offers a validated reverse vaccinology framework for future brucellosis vaccine development.

## 1. Introduction

Brucellosis, caused by facultative intracellular bacteria of the genus *Brucella*, is among the most widespread zoonoses worldwide. In endemic regions, its chronic and recurrent nature drives persistent economic losses and public-health challenges. As a facultative intracellular pathogen, *Brucella* survives and replicates within macrophages and other host cells, establishing long-term chronic infections that are difficult to eradicate. Protective immunity therefore depends chiefly on Th1-polarized cellular responses-IFN-γ-secreting CD4^+^ helper T (Th) cells and cytotoxic CD8^+^ cytotoxic T (Tc/CTL) cells together with durable immunological memory. Because protection is so heavily Th1-dependent, a vaccine must be a potent inducer of T-cell responses; this requirement helps explain why traditional single-antigen vaccines, which tend to favor humoral immunity, offer only limited protection [1,2,3,4,5,6].

Live attenuated vaccines are currently the mainstay for controlling brucellosis in livestock, but residual virulence and the risk of abortion preclude their use in humans [7,8,9,10]. Conventional single-antigen subunit vaccines, for their part, generally fail to elicit robust cellular immunity and therefore offer insufficient protection. Multi-epitope platforms can overcome both limitations: they engage humoral and cellular arms simultaneously while maintaining a favorable safety profile, making them a promising direction for brucellosis vaccine development [11,12,13].

In this strategy, immunodominant T- and B-cell epitopes from several conserved antigens are assembled in tandem, allowing humoral and cellular immunity to be induced together without the safety concerns associated with live attenuated strains. Compared with single-antigen subunits, multi-epitope constructs pool protective epitopes from multiple antigens to broaden coverage and can be tailored to the HLA profile of the target population, a feature especially useful for intracellular pathogens like *Brucella* [14].

Even so, two shortcomings have slowed the clinical translation of existing multi-epitope brucellosis vaccines. Nearly all published designs rest entirely on in silico prediction and lack in vivo validation of epitope immunogenicity in genuine infection models. In addition, most constructs are not optimized for population-specific HLA allele frequencies, so their coverage in the intended population is unpredictable.

Although multi-epitope *Brucella* vaccines have recently progressed to experimental expression, mouse immunization, and virulent challenge, including an extracellular-vesicle-guided multi-epitope construct protective against *B. abortus* [15] and a multi-epitope mRNA vaccine protective against *B. melitensis* [16], the integration of population-informed human HLA selection with experimental pre-screening of each predicted peptide before assembly remains underexplored.

To address these gaps, we built an integrated reverse vaccinology pipeline that combines bioinformatic prediction with in vivo immunogenicity validation. We selected two functionally complementary groups of conserved *Brucella* antigens as the epitope-mining pool and predicted MHC-I/II epitopes against high-frequency HLA alleles prevalent in the Chinese population. Candidate epitopes were then tested for immunogenicity in vivo by IFN-γ ELISpot using a chronic mouse infection model. The shortlisted candidate immunoreactive epitopes were assembled into a multi-epitope candidate named REC1, which we further characterized in silico for physicochemical properties, structural architecture, and simulated immune responses. This work provides both a methodologically validated framework and an early-stage candidate for brucellosis multi-epitope vaccine development.

REC1 is designed as a human vaccine; because no human brucellosis vaccine is currently licensed, its development will proceed through recombinant expression, HLA-matched immunogenicity studies, virulent-challenge protection studies in animal models, and subsequently the standard staged preclinical and clinical evaluation pathway.

## 2. Materials and Methods

### 2.1. Candidate Protein Selection and Characterization

We retrieved the amino acid sequences of six candidate antigens, GroEL, DnaK, Omp19, Omp31, Omp25, and BamA, from the *B. melitensis* strain 16M reference proteome in the UniProt database (https://www.uniprot.org/). Accession numbers for all six proteins are listed in Table 1 [17].

To determine whether these proteins could serve as potential vaccine antigens, we performed several in silico analyses of the candidate proteins. Functional annotation of all protein sequences was conducted via InterProScan (http://www.ebi.ac.uk/interpro/search/sequence-search (accessed on 6 May 2026)) [18], while functional interpretation was done through QuickGO (https://www.ebi.ac.uk/QuickGO/ (accessed on 6 May 2026)) [19]. We used the UniProt BLAST web tool (blast program, https://www.uniprot.org/blast/) (accessed on 8 May 2026) to assess sequence conservation against the reference proteomes of major *Brucella* species, namely *B. melitensis* 16M, *B. abortus* 2308, *B. suis* 1330, *B. ovis*, *B. microti*, *B. canis*, *B. pinnipedialis*, and *B. ceti*. Antigenicity was predicted via the VaxiJen server (http://www.ddg-pharmfac.net/vaxijen/VaxiJen/VaxiJen.html (accessed on 8 May 2026)) using the bacterial model with a 0.4 threshold; proteins exceeding this cutoff were classified as predicted protective antigens [20].

### 2.2. Screening of High-Frequency HLA Alleles in the Chinese Population

Chinese population HLA allele frequencies were retrieved from the Allele Frequency Net Database (AFND) [21]. For each reported allele, we used the “% of individuals” value, i.e., the population carrier frequency. Records with a carrier frequency of zero were removed, and for each allele the highest carrier frequency reported across Chinese study populations was retained, yielding 428 non-zero HLA class I records corresponding to 179 distinct allele labels and 561 non-zero HLA class II records corresponding to 124 distinct labels. Allele names were standardized to four-digit (two-field) resolution by collapsing higher-resolution subtypes to their four-digit parent allele in accordance with IMGT/HLA nomenclature [22]; the non-expressed null allele A*02:53N was retained in the audit count but excluded from functional coverage calculations. An operational carrier frequency cutoff of ≥3% was then applied, defining a prediction panel of 49 HLA class I alleles (15 HLA-A, 22 HLA-B, 12 HLA-C) and 71 HLA class II entries (37 DRB1, 8 DQA1, 15 DQB1, 11 DPB1) (Supplementary Tables S1 and S2). A 3% allele-frequency cutoff was applied to retain the common HLA alleles that afford broad population coverage, while excluding the large number of low-frequency alleles and thereby markedly reducing the number of proteome-wide pan-allele binding predictions at a minimal cost in coverage.

To verify empirically that the 3% cutoff was not arbitrary, we constructed frequency-ranked cumulative carrier coverage (rarefaction). For HLA class I as a whole and for each locus separately, alleles were ranked in descending order of carrier frequency and added sequentially, and cumulative carrier coverage after adding the n highest-frequency alleles was calculated as one minus the product of the individual non-carrier frequencies of those n ranked alleles. Because HLA class II antigen presentation is mediated by α/β heterodimers rather than by individual chains, DRB1, DQA1, DQB1 and DPB1 were analyzed separately and no cross-chain product was computed. The low-resolution label DQA1*03 was retained in the 71-entry screening panel but excluded from the four-digit accumulation, giving 70 non-overlapping four-digit class II panel restrictions (37 DRB1, 7 DQA1, 15 DQB1, 11 DPB1).

### 2.3. T-Cell Epitope Prediction and Filtering

Epitope prediction was performed on the Immune Epitope Database (IEDB) platform (https://www.iedb.org/) (Supplementary Tables S1 and S2) [23]. For MHC class I-restricted CTL epitopes, we ran NetMHCpan 4.1 and defined peptides with %Rank ≤ 0.5 as strong binders (SB). MHC class II-restricted Th epitopes were predicted with NetMHCIIpan 4.1, applying a %Rank ≤ 2 cutoff for the SB category [24].

For MHC class II prediction, each restriction was specified as the functional α/β heterodimer recognized by NetMHCIIpan. Because the HLA-DR α-chain (DRA) is essentially monomorphic, HLA-DR restrictions were entered as HLA-DRB1 alleles, whereas HLA-DQ and HLA-DP restrictions, whose peptide-binding specificity is defined by the pairing of both polymorphic chains, were entered as explicit α/β heterodimers (e.g., HLA-DQA1*01:02/DQB1*06:09 and DPA1*01:03/DPB1*04:02) [24,25].

Population coverage of the final incorporated epitope set was estimated for the China Oriental population using the IEDB Population Coverage tool (web version; http://tools.iedb.org/population/ (accessed on 28 September 2026)), which derives the fraction of individuals expected to respond to an epitope set from HLA genotypic frequencies under Hardy–Weinberg equilibrium [26]. For each incorporated epitope, all of its strong-binding restricting alleles were entered (Supplementary Table S3).

MHC-I coverage was calculated for the five CTL epitopes, HLA-DR coverage for the four DR-bearing Th epitopes using their DRB1 alleles, and a combined class I+II estimate was then generated. Because all HLA-DP restrictions of the BamA Th epitope were predicted with the fixed predominant DPA1*01:03 α-chain of HLA-DP4 [27,28], an extended class II and combined estimate was additionally obtained by entering the DPB1 alleles as a β-chain-level approximation. The IEDB tool does not accept paired DQA1/DQB1 (or DPA1/DPB1) heterodimers, and entering DQB1 chains alone would overestimate coverage, because HLA-DQ binding specificity is jointly determined by both polymorphic chains [25,29]. DQ component coverage was therefore estimated independently from population DQA1-DQB1 cis-haplotype (gametic) frequencies of the four Chinese ethnic groups (Han, Hui, Kazakh, Uyghur) for which complete two-locus DQA1-DQB1 haplotype data are available in AFND [21,30].

For each group, the summed frequency of target haplotypes, s, was converted to the fraction of individuals carrying at least one target haplotype as C = 1 − (1 − s)^2^ = 2s − s^2^ under Hardy–Weinberg equilibrium [26]; only cis-encoded pairings were counted, because trans-only DQ variants contribute minimally to the DQ immunopeptidome [31]. A low-resolution DQA1*03 record could not be assigned to a specific DQA1 allele and was therefore excluded; consequently, the estimated DQ coverage is a conservative lower bound and may underestimate coverage contributed by individuals carrying unresolved DQA1*03-bearing haplotypes.

Because HLA-DQ is in strong linkage disequilibrium with HLA-DRB1 and the DQ haplotype data derive from independent, smaller cohorts, DQ coverage was reported separately and was not merged into the combined IEDB estimates, which assume linkage equilibrium across loci.

### 2.4. IFN-γ ELISpot Epitope Validation in Brucella-Infected Mice

All experiments were conducted in an animal biosafety level 2 (ABSL-2) facility.

We used 8-week-old female BALB/c mice (*n* = 4 or 5 per group). Mice in the infection group received an intraperitoneal injection of 1 × 10^6^ CFU of *Brucella* strain M5-90Δ26 (a gift from the Hengliang Wang group, National Key Laboratory of Advanced Biotechnology, Academy of Military Medical Sciences, Beijing, China). M5-90Δ26 is a BP26-deletion derivative of the attenuated *B. melitensis* vaccine strain M5-90 and was used to establish a chronic *Brucella* infection in mice, in which the predicted peptides were tested by IFN-γ ELISpot [32]. Mice were healthy; infection was confirmed by splenomegaly and recall.

All mice were monitored daily for general health, behavior, body weight, and signs of illness; no unexpected adverse events were observed, and humane endpoints were applied in accordance with the approved protocol. Establishment of infection before harvesting was confirmed by splenomegaly and the robust, antigen-specific recall responses in infected animals, whereas PBS-control mice remained negative throughout; splenic CFU was not separately enumerated in this screening study, and inter-individual variation is reported as mean ± SD with individual-animal points.

This attenuated strain was chosen because it can be handled at ABSL-2 while still establishing a chronic, disseminated infection with persistent splenic bacterial load, providing a genuine infection context in which to test whether predicted peptides are processed and presented strongly enough to prime peptide-specific T-cells, without the biosafety burden of virulent challenge at the screening stage. It serves strictly as the infection model, not as the vaccine candidate (REC1 is the candidate); its BP26 deletion additionally supports DIVA, and because no REC1 epitope is derived from BP26, the deletion does not remove any antigen under study. The corresponding limitation, that an attenuated strain may not fully reproduce virulent infection, is addressed in Section 4.

Control littermates received the same volume of sterile PBS by the same route. Twenty-eight days after infection, we isolated splenocytes from each mouse and plated 2 × 10^5^ cells per well in ELISpot plates (Dakewe Biotech, Shenzhen, China). Each well was stimulated with one synthetic peptide at 1 μg/well (in 10 μL). The peptides were synthesized by BBI (Shanghai, China) at >98% purity and dissolved in PBS at 2 mg/mL. PMA plus ionomycin (Dakewe Biotech, China) and complete medium alone were included as positive and negative controls, respectively.

Each peptide was assayed in one well per individual mouse. The individual mouse was the experimental unit: each animal contributed a single net SFU value per peptide, and all between-group tests were performed on these animal-level values (*n* = 5 infected vs. *n* = 5 control for the MHC-I panel; *n* = 4 vs. *n* = 4 for the MHC-II panel), so no pseudoreplication was present [33].

Spot-forming unit (SFU) counts were normalized to 10^6^ splenocytes. When the SFU (after subtraction of the negative control value) was negative, we considered this value to be zero.

Quality control criteria: (i) the SFU of the negative control well was required to be <40 per 10^6^ splenocytes for each mouse; (ii) the positive control well was considered valid if its SFU was ≥5-fold the negative control SFU of the same mouse and ≥25 per 10^6^ splenocytes. Mice that failed QC were excluded from all peptide analyses.

For every single mouse, a peptide-specific response was defined as positive when both: (a) SFU ≥ twofold that of the corresponding negative control, and (b) net SFU (peptide SFU minus negative control SFU) ≥50 per 10^6^ splenocytes. Responder rate for each peptide was determined by dividing the number of responder mice by the number of QC-passed mice in the infected group. QC criteria were applied to infected and control mice prior to comparing groups [34,35,36]. Responder proportions are reported with exact two-sided 95% confidence intervals (95% CI) [37], which are necessarily wide at these small sample sizes and are provided to make the uncertainty in responder frequency explicit. The individual-animal positivity rule and the ≥50% responder rate screening threshold were defined before data analysis. The decision to use responder rate as the primary selection criterion (rather than multiplicity-adjusted *p* values) was made after observing that the discrete exact test at these sample sizes could not attain Bonferroni-adjusted significance.

For each peptide, net SFUs were compared between *Brucella*-infected and PBS-control mice using an exact two-tailed Mann–Whitney U test, with *p* values Bonferroni-corrected within two pre-defined comparison families analyzed separately (the 13 MHC-I peptides and the 6 MHC-II peptides). Because of the very small group sizes, the exact test has a discrete lower bound: even with complete group separation, the smallest attainable two-sided *p* was 0.0079 for 5-vs-5 (MHC-I) and 0.0286 for 4-vs-4 (MHC-II), giving Bonferroni-adjusted floors of 0.1032 and 0.1714, respectively. Accordingly, no peptide reached an adjusted *p* < 0.05. We therefore used the pre-specified responder rate cutoff (≥50% of QC-passed infected mice responding) as the primary, exploratory criterion to shortlist candidate immunoreactive epitopes, while the unadjusted group-level *p* values and effect sizes were treated as supportive descriptive evidence. This procedure is intended for candidate discovery, not confirmatory hypothesis testing.

### 2.5. Bioinformatic Comparison of Candidate Carrier Proteins

Three endogenous proteins from *Brucella melitensis* biotype 1 strain 16M were selected as candidate vaccine carriers: ribosomal protein L7/L12, outer membrane protein Omp28 (BP26), and lumazine synthase BLS. Their amino acid sequence information can be found in the UniProtKB database under UniProt accession numbers P0A468, P0A3U8, and Q8YGH2, respectively.

We computed basic physicochemical parameters on the ExPASy ProtParam server (https://web.expasy.org/protparam/ (accessed on 1 July 2026)), using an instability index of 40 as the stability threshold. Proteins scoring below this value are regarded as stable [38]. Antigenicity was predicted on the VaxiJen v2.0 server with the bacterial protein model.

To verify that AAY linkers would be properly cleaved by the proteasome, we ran NetChop 3.1 (C-term 3.0 model, threshold = 0.5) (https://services.healthtech.dtu.dk/service.php?NetChop-3.1 (accessed on 1 July 2026)) [39].

### 2.6. In Silico Prediction of Allergenicity, Antigenicity, and Toxicity of the Constructed Vaccine Candidate

We evaluated the potential allergenicity of REC1 in silico using three independent servers: AllerTOP v2.1, AllergenFP v1.1, and AlgPred 2.0 [40].

For AlgPred 2.0, overall allergenic potential was predicted via the Prediction of Allergens module (https://webs.iiitd.edu.in/raghava/algpred2/batch.html (accessed on 5 August 2026)) using the hybrid (RF + BLAST + MERCI) ensemble model at a classification threshold of 0.3. Linear IgE epitope mapping was conducted against experimentally validated IgE epitopes from the IEDB using the IgE epitope Mapping module (https://webs.iiitd.edu.in/raghava/algpred2/ige_2.html (accessed on 5 August 2026)), and conserved IgE-associated allergenic motifs were scanned via the Motif Scanning module (https://webs.iiitd.edu.in/raghava/algpred2/ige.html (accessed on 5 August 2026)) with the MERCI algorithm. Homology-based BLAST searches were performed using the BLAST for Similarity Search module (https://webs.iiitd.edu.in/raghava/algpred2/blast.html (accessed on 5 August 2026)) against both the Allergen and Non-allergen Database and the IgE epitope Database, with an E-value cutoff of 1 × 10^−6^.

Protein toxicity was assessed with ToxinPred 2.0 (https://webs.iiitd.edu.in/raghava/toxinpred/ (accessed on 9 August 2026)), and full-length REC1 protective antigenicity was re-evaluated via VaxiJen.

### 2.7. Epitope-Level Sequence Conservation Analysis

For each species, the full-length ortholog with the highest sequence identity was retained; hits corresponding to distinct paralogs (e.g., Omp31b/Omp31-1) were recorded separately and not treated as the source antigen ortholog. The start and end coordinates of each shortlisted epitope were located on the source antigen, and the corresponding residues of each retrieved homolog were read directly from the pairwise alignment. An epitope was classified as fully conserved (100%) when every residue was identical to the *B. melitensis* 16M sequence, as substituted when one or more residues differed (with positions and changes recorded), or as absent when no source antigen ortholog was present.

Predicted MHC-binding anchor and TCR-facing positions were assigned within the NetMHCpan/NetMHCIIpan 9-mer core. For class I, the canonical anchors are P2 and the C-terminal PΩ [41,42]. For class II, the canonical anchors occupy the P1, P4, P6 and P9 pockets of the open-ended groove [43,44]. The remaining, upward-pointing (solvent-exposed) core residues, and, for class I, residues that bulge out of the groove because the termini are fixed, were assigned as the predicted TCR-facing region [45,46]. Allele-specific anchor exceptions, P3 in HLA-A*01, P5 in HLA-B*08, and P1 in HLA-A*26, were taken from the predictor and from large-scale ligand/structural analyses [47,48]. All assigned anchor and TCR-facing positions are predicted, not experimentally mapped.

### 2.8. In Silico Physicochemical and Structural Characterization

We used ExPASy ProtParam to derive the basic physicochemical parameters of REC1. The PSIPRED online server (https://bioinf.cs.ucl.ac.uk/psipred/ (accessed on 26 July 2026)) was used to predict the α-helix, β-strand, and random-coil contents for each REC1 domain [49]. The SWISS-MODEL platform (https://swissmodel.expasy.org/) [50] was used for automated homology modeling, generating three candidate 3D structures. The optimal model was selected based on the QMEANDisCo global score. The Ramachandran plot obtained from the SWISS-MODEL structure assessment was used to evaluate backbone dihedral stereochemistry of the template-aligned structural domain. Intrinsic disorder was predicted using AIUPred v2 (https://iupred.elte.hu/) [51] with a threshold of 0.5 (scores > 0.5 indicate a disordered region). Metapredict V3 (v3.1.0), a deep-learning-based consensus predictor of protein disorder, was employed to profile intrinsically disordered regions and simulate AlphaFold2-style per-residue predicted local distance difference test (pLDDT) scores across the full-length vaccine candidate sequence. The default metapredict V3-2024 disorder network and pLDDT V2-2024 network were applied with built-in thresholds. The boundaries and mean disorder scores of all identified intrinsically disordered regions (IDRs) were extracted for subsequent structural characterization (https://metapredict.net/) [52,53]. The full-length REC1 monomer (357 aa) was additionally modeled de novo with AlphaFold 3 (25 models across five seeds) [54]; the highest-ranked model was retained, with per-residue pLDDT and PAE, and geometry was validated with MolProbity [55].

### 2.9. In Silico Human Immune Simulation via C-ImmSim

We employed the C-ImmSim server (https://kraken.iac.rm.cnr.it/C-IMMSIM/ (accessed on 14 July 2026)) to model the human immune response elicited by REC1 and to assess its intrinsic immunogenicity under optimized simulation conditions [56,57,58].

Due to the allele input limit of the server, two parallel simulations were conducted with distinct HLA-B allele pairs, Simulation 1: HLA-B*15:01/HLA-B*54:01; Simulation 2: HLA-B*15:02/HLA-B*58:01, while maintaining identical HLA-A*01:01/HLA-A*01:01 homozygous and HLA-DRB1*10:01/HLA-DRB1*15:01 backgrounds. This design ensured that each CTL epitope and each DRB1-restricted Th epitope included in REC1 was represented in at least one simulation. The two HLA-B haplotype combinations were selected from the high-frequency allele panel of the Chinese population to ensure full coverage of all five CTL epitopes included in REC1; that is, each CTL epitope was covered by at least one allele in the selected panel. The antigen dose of 1000 molecules per injection is a standardized parameter of the C-ImmSim platform for qualitative immune trend simulation. The absolute values of simulation outputs do not directly reflect in vivo immune magnitudes and should be interpreted as kinetic trends rather than quantitative predictions.

In both simulations, the following global parameters were fixed: random seed = 12,345; Simulation Volume = 10; Total Simulation Steps = 350 (each step represents 8 h in vivo, total observation period approximately 117 days). The immunization schedule was a homologous prime-boost regimen with injections at day 0 (step 1), day 14 (step 43), and day 28 (step 85). Each injection consisted of 1000 antigen molecules administered with a uniform adjuvant strength of 100 (a standard setting to ensure full innate immune activation), and no LPS was included.

After every simulation run, dynamic curves of B-cells, Th-cells, CTL-cells, memory lymphocytes, and key effector cytokines were exported for quantification.

Due to the input limitations of the C-ImmSim server, only HLA-A, -B, and -DRB1 alleles were explicitly specified; other classical HLA loci (HLA-C, -DP, -DQ) were left as the platform’s default (unspecified), which may not fully represent antigen presentation via these molecules. Furthermore, *Brucella* lipopolysaccharide (LPS) was not included in the simulation, as our goal was to evaluate the intrinsic immunogenicity of the proteinaceous REC1 antigen; thus, the simulation does not capture the possible immunomodulatory effects of LPS-mediated innate signaling.

### 2.10. Molecular Docking and Interface Analysis

Full-length REC1, its individual modules (the L7/L12 carrier, CTL array, and Th array), and the five mature source antigens were docked against the ectodomains of human TLR2 (PDB 2Z7X) and human TLR4/MD-2 (PDB 3FXI). An extended panel of representative innate sensors was also examined: CD14 (PDB 1WWL, murine), the C1q globular head (PDB 2JG8), and the DC-SIGN carbohydrate-recognition domain (PDB 1K9I). Docking was performed with HADDOCK 3 in two modes: an unrestrained (ab initio) mode with only center-of-mass restraints, and a pocket-directed (ambiguous interaction restraint, AIR) mode in which receptor active residues were taken exclusively from experimental ligand contacts and ligand active residues were defined by relative solvent accessibility (≥0.30) [59]. The top three clusters were retained per group and mode. Complexes were analyzed with in-house contact analysis implementing the COCOMAPS 2.0 distance cutoffs (contacts ≤ 8 Å; hydrogen bond donor-acceptor ≤ 3.5 Å; salt bridges ≤ 4.0 Å; hydrophobic contacts ≤ 5.0 Å; π-type contacts ≤ 6.0 Å), reporting buried surface area (BSA), hydrogen bonds, salt bridges, hydrophobic and π contacts. The analysis was not performed on the COCOMAPS 2.0 server [60,61]. Hydrogen bond counts in the interface table are atom-pair distances ≤ 3.5 Å without an angle criterion (upper estimates), whereas contact maps count the shortest N-O distance between residue pairs. A pre-set low-confidence interface fraction (fraction of interface residues with pLDDT < 70) of 0.20 distinguished well-defined interfaces from hypothesis-generating contacts.

### 2.11. Representative Peptid–-MHC Modeling

A representative peptide–MHC (C1, LQKMKETAESY, bound to HLA-B*15:01) was constructed by template replacement from the same-allele experimental structure PDB 1XR9. The P1–P2 and P10–P11 anchors were positioned on the template anchor geometry, and the central P3–P9 loop was closed by Monte Carlo torsion sampling, side-chain repacking, and restrained FastRelax with the MHC backbone fixed. Anchor geometry, peptide–MHC hydrogen bonds, steric clashes, and anchor backbone RMSD versus the template were reported. TCR docking was not performed because a meaningful TCR-pMHC model requires a matched TCR clonotype sequence or an experimental template for the same peptide–MHC pair, neither of which was available.

### 2.12. Statistical Analysis

Statistical analyses were performed in GraphPad Prism v10.1.2. For IFN-γ ELISpot, peptide-level infected-versus-control comparisons used exact Mann–Whitney U tests on animal-level data, with Bonferroni correction applied within the MHC-I (13-test) and MHC-II (6-test) families; both unadjusted and adjusted *p* values are reported. All ELISpot epitope selection was exploratory and based on the pre-specified responder rate threshold.

## 3. Results

### 3.1. Epitope Prediction Profiles of Candidate Proteins

All six candidate proteins exceeded the 0.4 VaxiJen threshold and were classified as predicted protective antigens, and they were therefore included in the subsequent epitope prediction workflow. Except for Omp31, which is naturally absent from *B. abortus* owing to a large genomic deletion, all candidate antigens showed >97% sequence identity across the major pathogenic *Brucella* species (*B. melitensis* 16M, *B. abortus* 2308, *B. suis* 1330, *B. ovis*, *B. microti*, *B. canis*, *B. pinnipedialis*, and *B. ceti*). Detailed conservation data are provided in Supplementary Table S4. The absence of Omp31 in *B. abortus* is consistent with previous reports [62,63].

We ultimately selected 49 HLA class I alleles and 71 HLA class II alleles (Supplementary Tables S1 and S2), providing broad population coverage for downstream epitope prediction and vaccine design. This panel also supplied the homozygous HLA alleles used in the immune simulations. These allele frequencies characterize the initial screening panel and are not equivalent to the coverage achieved by the final construct; the predicted population coverage of the incorporated epitope set is reported in Section 3.4 and Table S5.

Applying the ≥3% carrier frequency cutoff retained 49 of 179 distinct non-zero HLA class I allele labels and 71 of 124 HLA class II labels; the 130 excluded class I labels comprised 129 low-frequency functional alleles and one non-expressed null allele (A*02:53N) (Table S6). Frequency-ranked cumulative carrier coverage curves showed that the cutoff coincided with a clear saturation plateau (Figure S1). For HLA class I, the 49-allele panel already achieved 99.90% cumulative carrier coverage, whereas adding all 178 functional positive alleles raised coverage only to 99.95%, an absolute long tail gain of just 0.05 percentage points (pp); locus-specific long tail gains were likewise small for HLA-A (15 panel versus 46 total alleles; 90.80% to 91.67%, +0.87 pp), HLA-B (22 versus 88; 89.70% to 92.54%, +2.83 pp) and HLA-C (12 versus 44; 89.04% to 91.20%, +2.16 pp), with cross-locus complementarity accounting for the near-complete combined coverage. Among HLA class II loci, the panels were similarly near saturation for DRB1 (37 panel versus 66 four-digit alleles; 99.51% to 99.57%, +0.07 pp), DQA1 (7 versus 7; 91.99%, with no four-digit long tail) and DQB1 (15 versus 27; 97.85% to 97.94%, +0.09 pp); DPB1 showed the largest, although still modest, long tail gain (11 versus 23; 87.49% to 88.94%, +1.45 pp). These single-chain curves describe the screening alleles rather than functional heterodimers, and the final REC1 epitope set nonetheless reached 96.10% predicted coverage for the five Th epitopes (HLA-DR plus HLA-DP) and 98.88% combined class I/II coverage in the China Oriental population (Section 3.4), indicating that the residual DPB1 long tail does not materially limit epitope set coverage. Taken together, these results demonstrate that the 3% cutoff captures the saturation inflection point of Chinese HLA diversity while excluding more than 70% of low-frequency class I labels at a cumulative coverage cost of only 0.05 pp.

Table 2 summarizes, for each of the six antigens, the total number of predicted MHC-I and MHC-II epitopes, the number remaining after deduplication, and the number of strong binders (SB). The SB counts in this table use standard cutoffs (%Rank ≤ 0.5 for MHC-I and %Rank ≤ 2 for MHC-II) to give a broad picture of the binding landscape; note that the peptides later chosen for experimental testing met more stringent criteria (detailed below). Because our allele panel covers 49 HLA class I and 71 HLA class II alleles prevalent in the Chinese population, the total number of predicted epitopes is correspondingly large.

### 3.2. IFN-γ ELISpot Validation in Brucella-Infected Mice

To identify epitopes recognized by T-cells during natural *Brucella* infection, we selected high-percentile-rank peptides from the epitope pools (Table 3) and tested their immunoreactivity by IFN-γ ELISpot, restimulating splenocytes from *Brucella*-infected BALB/c mice with each synthetic peptide. The 19 synthetic peptides evaluated are listed in Table 3. All mice passed QC. In the PBS control group, no peptide met the pre-defined individual mouse positivity criteria.

Peptide-level comparisons used exact two-sided Mann–Whitney tests with Bonferroni correction (13-test MHC-I and 6-test MHC-II families; Table 4). At these small sample sizes, the exact test has a discrete lower bound, so even complete group separation could not yield adjusted *p* < 0.05 (best adjusted *p* = 0.1032 for MHC-I, 0.1714 for MHC-II). We therefore shortlisted peptides using the pre-specified responder rate threshold (≥50%), with unadjusted *p* values serving as supportive descriptive evidence.

**Figure 1:**
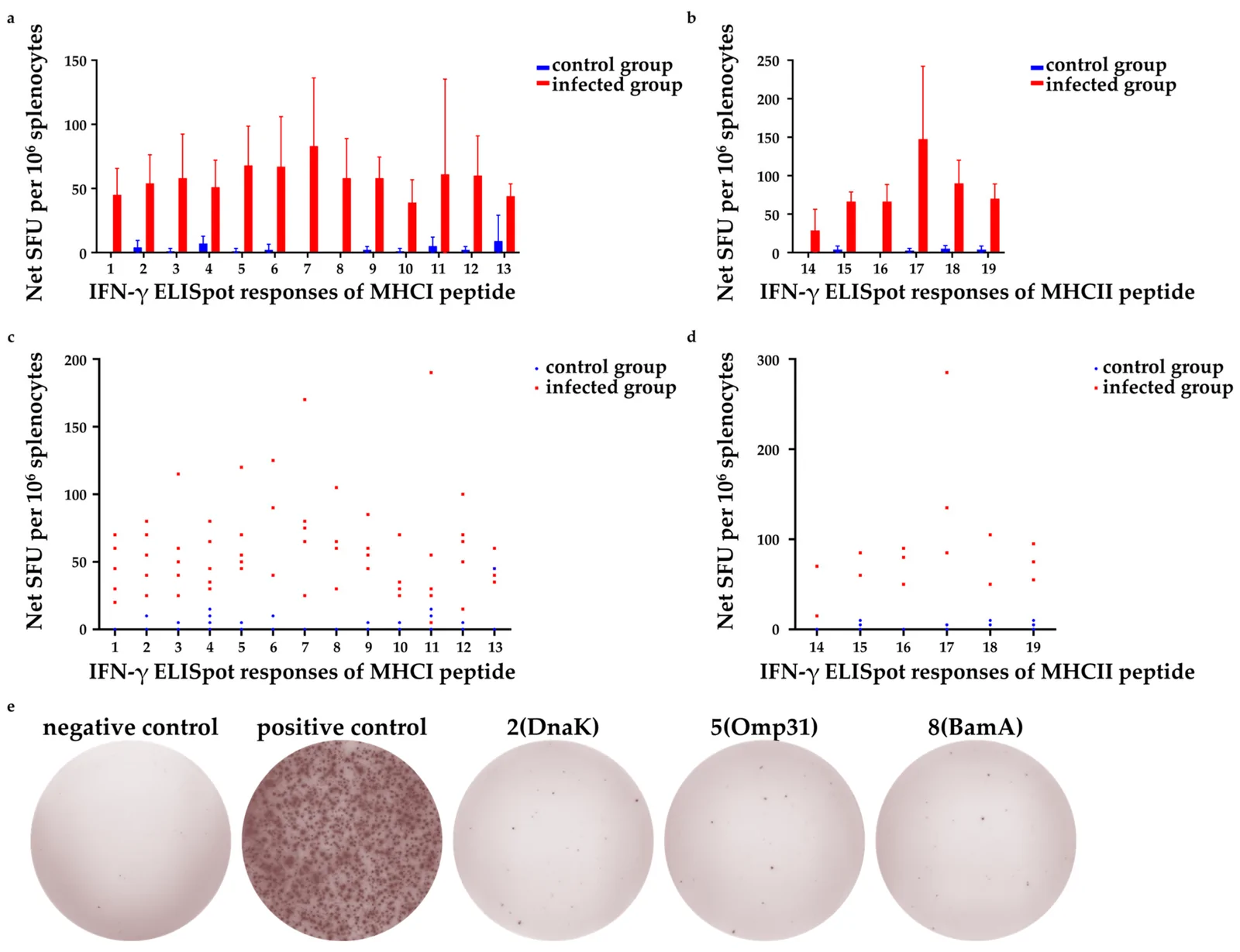
IFN-γ ELISpot responses in splenocytes from PBS-control and *Brucella*-infected mice (*n* = 5 per group for MHC-I, *n* = 4 per group for MHC-II; one well per peptide per animal; the individual mouse was the experimental unit): (**a**) Mean net SFU per 10^6^ splenocytes (mean ± SD) for MHC-I peptides (1–13); (**b**) corresponding data for MHC-II peptides 14–19; (**c**,**d**) individual-animal net SFU corresponding to (**a**,**b**); (**e**) representative wells per 2 × 10^5^ cells: negative control, positive control, and peptides 2, 5, and 8. Peptide-level infected-versus-control comparisons used exact two-sided Mann–Whitney tests with Bonferroni correction within the MHC-I (13) and MHC-II (6) families. No comparison reached Bonferroni-adjusted *p* < 0.05 (best adjusted *p* = 0.1032 for MHC-I and 0.1714 for MHC-II, bounded by the discrete test distribution at these sample sizes); no significance is shown. Peptides were shortlisted as candidate immunoreactive epitopes using the pre-specified ≥50% responder rate threshold (Table 4).

The responder rates, net SFU per 10^6^ splenocytes, and adjusted *p* values from Mann–Whitney *U* tests are presented in Table 4. Quantitative group comparisons are displayed as bar charts in Figure 1a,b, while individual net SFU values for each mouse are shown as scatter plots in Figure 1c,d. Representative ELISpot well images, including positive and negative control wells and representative experimental wells (2 × 10^5^ cells/well), are presented in Figure 1e.

Using the pre-specified ≥50% responder rate screen, seven MHC-I peptides (2, 3, 5, 7, 8, 9, 12) and five MHC-II peptides (15–19) were shortlisted as candidate immunoreactive epitopes. Several non-shortlisted peptides showed unadjusted group-level differences but responder rates below 50%; these differences did not survive Bonferroni correction (adjusted *p* ≥ 0.1032). Taken together, these data indicate that the shortlisted peptides are immunoreactive during chronic *Brucella* infection and can be recognized by infection-generated T-cells.

Of note, these experiments test peptide recognition after *Brucella* infection; mice were not immunized with REC1, and REC1 was not expressed or administered in this study. The data therefore validate the immunoreactivity of the individual peptides, not the immunogenicity of the assembled REC1 construct, which remains to be evaluated.

Taken together, these responses demonstrate that T-cells generated during *Brucella* infection can recognize the synthetic peptides in an H-2d context. They do not, however, establish two points: presentation by the predicted human HLA alleles, and efficient processing and presentation of these sequences from the assembled REC1 construct, because exogenous synthetic peptide bypasses cellular processing. The former requires HLA-restricted testing in HLA-transgenic mice or human cells, and the latter requires direct processing-and-presentation assays after REC1 expression.

### 3.3. Screening and Identification of the Optimal Vaccine Carrier Backbone

To determine an appropriate carrier domain for our modular subunit vaccine, we systematically compared the physicochemical properties and antigenic characteristics of three classical *Brucella*-derived candidate proteins (Table 5).

Each of the three candidate carriers had an instability index below 40, which suggests that all should be structurally stable when expressed recombinantly. Among them, L7/L12 scored the lowest (18.55), implying better intrinsic stability and less susceptibility to degradation during expression and purification. In VaxiJen predictions, all three were classified as potential protective antigens; L7/L12 received the highest score (0.6244), ahead of Omp28 (0.5381) and BLS (0.4891).

L7/L12 is also the smallest of the three (124 aa, ~12.55 kDa), minimizing steric hindrance with the fused target antigen and easing recombinant expression. Moreover, published work shows that L7/L12 exerts a strong Th1-biased adjuvant effect, a property critical for inducing cellular immunity against intracellular pathogens like *Brucella* [64,65,66,67].

Overall, L7/L12 surpassed Omp28 and BLS in structural stability, antigenicity, molecular size, and immunomodulatory function, and was chosen as the carrier backbone for the modular vaccine.

NetChop prediction confirmed high-confidence proteasomal cleavage sites at the C-terminus of tyrosine in all AAY linkers (prediction scores > 0.7) to enable efficient release of individual CTL epitopes.

### 3.4. Design and Construction of the REC1 Multi-Epitope Vaccine Candidate

Of the seven MHC-I and five MHC-II peptides shortlisted as candidate immunoreactive epitopes by ELISpot, one MHC-I and one MHC-II epitope from each of the five selected antigens were incorporated, yielding five CTL and five Th epitopes in REC1; the two additional shortlisted MHC-I peptides (both BamA-derived) were not incorporated because BamA was already represented by a single CTL epitope, thereby keeping one CTL and one Th epitope per antigen. The CTL array was placed upstream of the Th array to facilitate proteasomal processing before lysosomal compartmentalization, and the rigid EAAAK linker was used to maintain domain independence (peptide sequences in Table 6; the complete strong-binding HLA restriction profile of each incorporated epitope is provided in Supplementary Table S3, with DQ and DP entries written as functional α/β heterodimers).

**Figure 2:**
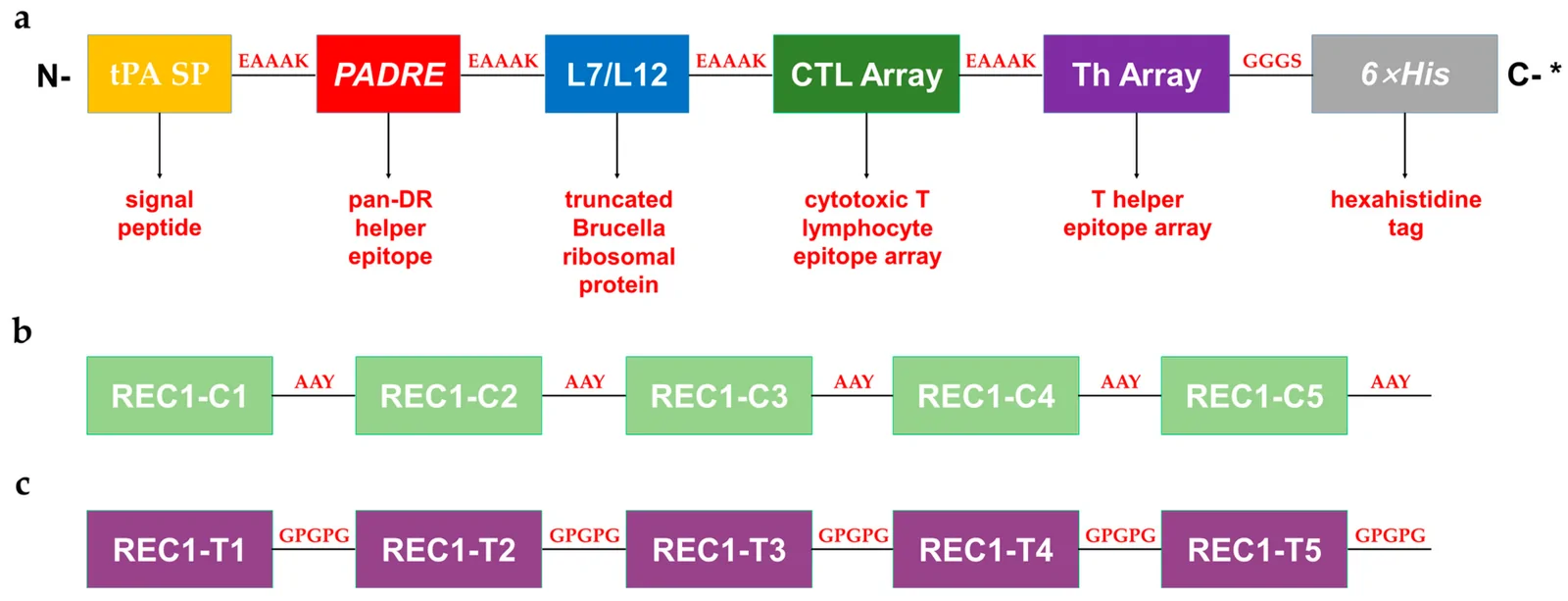
Schematic illustration of the REC1 multi-epitope vaccine candidate construct: (**a**) Linear domain architecture from N- to C-terminus. Functional modules are color-coded and connected by linkers as indicated. SP, signal peptide; PADRE, pan-DR helper epitope; L7/L12, truncated *Brucella* ribosomal protein; CTL, cytotoxic T lymphocyte epitope array; Th, T helper epitope array; 6×His, hexahistidine tag. EAAAK and GGGS denote rigid and flexible linkers, respectively; AAY and GPGPG indicate proteasomal and lysosomal cleavage sites, respectively; (**b**) detailed composition of the CTL epitope array with its respective linkers; (**c**) detailed composition of the Th epitope array with its respective linkers. The asterisk (*) at the C-terminus in panel (**a**) denotes the translation stop codon.

From N- to C-terminus, REC1 comprises a tPA secretion signal peptide (MDAMKRGLCCVLLLCGAVFVSPS), which promotes secretion and enhances uptake by antigen-presenting cells; an EAAAK linker; the PADRE universal pan-DR helper T-cell epitope (AKFVAAWTLKAAA) to broaden HLA restriction; another EAAAK linker; a truncated *Brucella* L7/L12 ribosomal protein carrier (ADLAKIVED-LSALTVLEAAELSKLLEEKWGVSAAAPVAVAAAGGAAPAAAAEEKTEFDVVLADG-GANKINVIKEVRALTGLGLKEAKDLVEGAPKAVKEGASKDEAEKIKAQLEAAGAKVELK) which serves as an endogenous Th1 immunostimulant, prior work has shown that L7/L12 intrinsically drives robust Th1-biased responses and acts as a self-adjuvant carrier that amplifies epitope-specific T-cell immunity when fused to target epitopes [8,68]; an EAAAK linker; the MHC-I CTL epitope tandem array, with AAY linkers inserted between CTL epitopes and at the C-terminus; an EAAAK rigid spacer separating the CTL and Th modules; the MHC-II Th tandem array, with GPGPG linkers between epitopes and at the C-terminus; a GGGS flexible linker; and a C-terminal 6×His tag for purification (Figure 2). At this stage, REC1 is an in silico-designed sequence assembled computationally as described above; it has not yet been physically synthesized or expressed, and gene synthesis, recombinant expression, and purification will be performed in subsequent work.

VaxiJen classified REC1 as a probable antigen, supporting its suitability as a vaccine immunogen.

AllerTOP v2.1 classified REC1 as a probable non-allergen. AllergenFP v1.1 and the AlgPred 2.0 hybrid model returned positive calls, but all sequence-homology-based assessments, IgE epitope mapping, MERCI motif scanning, and BLAST searches against allergen databases, yielded no significant matches. Such positive calls are widely recognized as low-specificity false positives, often triggered by the high Ala/Gly content of the REC1 sequence. Under the FAO/WHO allergenicity assessment framework, the primary risk criteria are sequence homology and matches to known IgE epitopes, rather than physicochemical feature similarity.

ToxinPred 2.0 classified REC1 as a non-toxic protein. None of the four component metrics flagged toxicity: the ML score was 0.26, the MERCI score was 0, the BLAST score was −0.5, and the composite score was −0.24. Specifically, a MERCI score of zero means no conserved toxic peptide motifs were found, and the negative BLAST score reflects a lack of sequence homology to known toxic proteins in the reference databases.

Predicted population coverage of the final REC1 epitope set. Using the IEDB Population Coverage tool for the China Oriental population, the five incorporated CTL epitopes (with all of their SB class I restrictions) were predicted to cover 71.39% of individuals (mean 1.37 epitope-HLA hits per person; PC90 = 0.35), and the DRB1 restrictions of the four DR-bearing Th epitopes covered 69.15% (1.50 hits; PC90 = 0.32). The combined class I + HLA-DR set, the most conservative estimate, requiring no assumption about paired class II heterodimers, reached 91.17% coverage (2.86 hits; PC90 = 1.09), indicating that more than 90% of individuals would recognize at least one incorporated T-cell epitope. Adding the DPB1 restrictions of the BamA Th epitope as a β-chain-level approximation (α-chain fixed to DPA1*01:03) raised class II coverage to 96.10% (2.69 hits; PC90 = 1.32) and the combined class I + II estimate to 98.88% (4.05 hits; PC90 = 2.17), an extended upper bound (Table S5).

Because the IEDB tool cannot model paired DQA1/DQB1 heterodimers, the DQ component was estimated separately from Chinese DQA1-DQB1 cis-haplotype frequencies under Hardy–Weinberg equilibrium (C = 2s − s^2^). Across the four Xinjiang ethnic groups, strict four-digit DQ coverage ranged from 57.75% (Hui) to 79.04% (Uyghur), with a value of 68.17% in Han Chinese, the group genetically closest to China Oriental; assigning low-resolution DQA1*03 records to DQA1*03:01 in a relaxed sensitivity analysis gave an upper-bound range of 71.00–84.99% (Han 81.02%) (Table S7). Owing to strong DR-DQ linkage disequilibrium and the independent, smaller DQ cohorts, DQ coverage is reported separately rather than merged into the combined, linkage-equilibrium-based IEDB estimates.

### 3.5. Epitope-Level Sequence Conservation Across Major Brucella Species

Because high whole-protein sequence identity does not guarantee conservation of an individual epitope, we extended the protein-level analysis to the epitope level. The residues corresponding to each of the 12 shortlisted candidate immunoreactive epitopes, and their predicted MHC anchor and TCR-facing positions, were read directly from the pairwise alignments (Tables S8 and S9).

All twelve epitopes were 100% identical across every species in which their source antigen ortholog was present, and every predicted MHC anchor and TCR-facing residue was likewise conserved in every species where the source antigen was present. Although the whole-protein identity of some proteins was slightly below 100%, every amino acid difference mapped outside the epitope regions.

The two Omp31-derived epitopes (C4, T4) were 100% conserved across *B. melitensis*, *B. suis*, *B. ovis*, *B. microti*, *B. canis*, *B. pinnipedialis*, and *B. ceti*. Consistent with the known genomic deletion spanning Omp31 [63], no Omp31 ortholog was identified in *B. abortus*, and C4 and T4 are therefore absent from the Omp31 protein in this species. Notably, a distinct, conserved paralog (Omp31b/Omp31-1, 65.2% identity present in *B. abortus*) encodes both the C4 and T4 sequences with 100% identity; this represents a potential but experimentally untested cross-reactivity and is not equivalent to the *Omp31* gene.

These data establish broad epitope-level rather than solely whole-protein conservation: the ten non-Omp31 epitopes are fully conserved in *B. abortus*, and the Omp31 epitopes are conserved across all other principal zoonotic species. Cross-species coverage claims are therefore based on epitope-level evidence.

### 3.6. In Silico Physicochemical and Structural Characterization of REC1

Table 7 lists the predicted physicochemical parameters of REC1. The construct is 357 amino acids long, with a theoretical molecular weight of 35.86 kDa and a pI of 5.64 (weakly acidic). An instability index of 25.25 points to high stability, and the aliphatic index (79.89) together with a GRAVY value of −0.015 confirms that the protein is hydrophilic and unlikely to aggregate. Predicted half-lives, 30 h in mammalian reticulocytes (in vitro), >20 h in yeast (in vivo), and >10 h in *Escherichia coli* (in vivo), indicate that REC1 is stable in both prokaryotic and eukaryotic contexts and is therefore well suited to recombinant expression in *E. coli*.

Analysis of the amino acid composition showed that alanine (23.0%) and glycine (12.6%) are the most abundant residues; both are overrepresented because of the L7/L12 carrier domain and the synthetic linkers. There are 38 negatively charged residues (Asp + Glu) versus 32 positively charged ones (Arg + Lys), which accounts for the acidic pI. Notably, cysteine makes up only 0.8% of the sequence, a low level that should minimize unwanted disulfide crosslinking during expression and purification.

**Figure 3:**
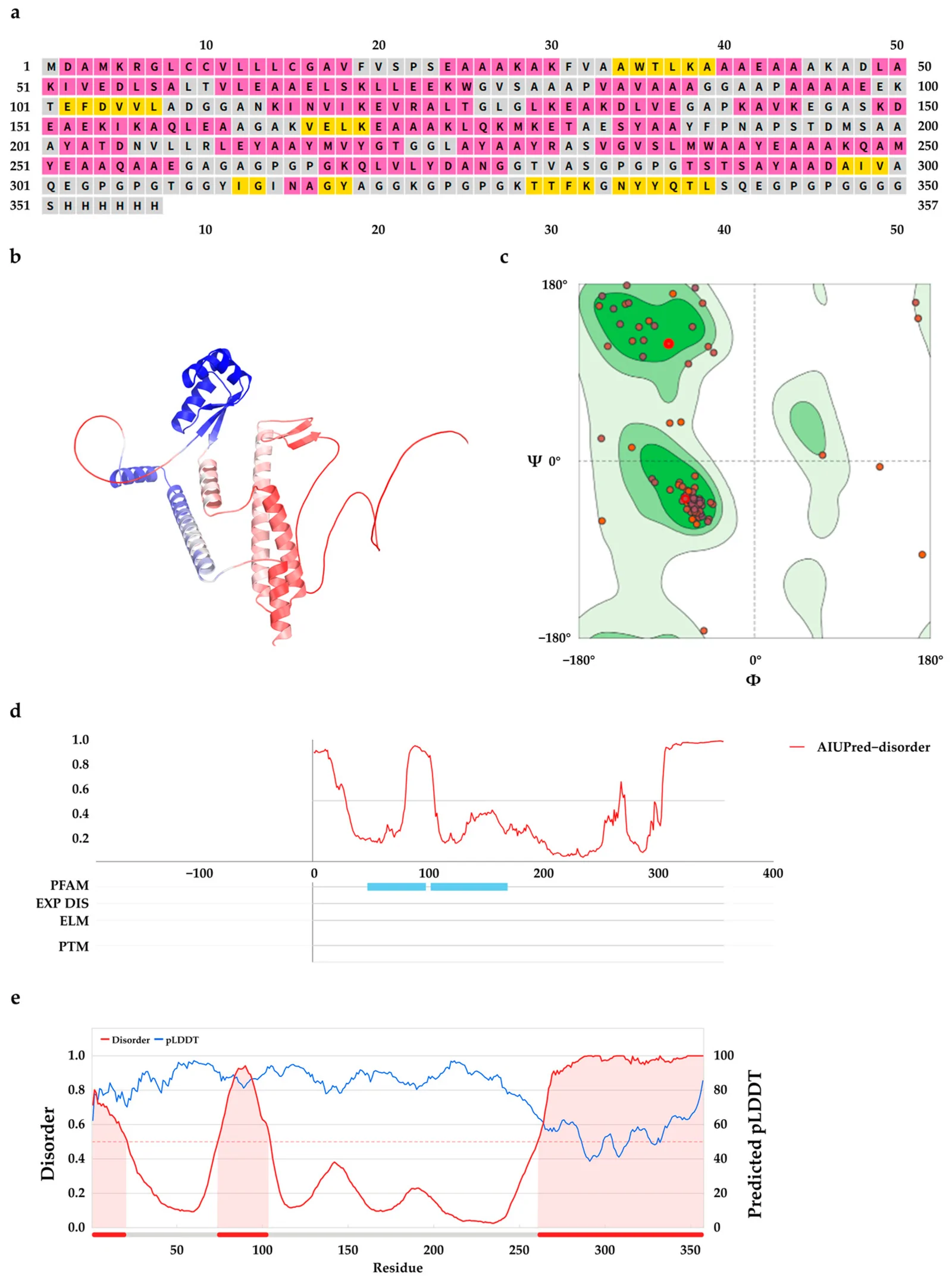
In silico structural characterization of the REC1 multi-epitope vaccine candidate construct: (**a**) Full-length secondary structure profile predicted by PSIPRED. Residues are color-coded by secondary structure type: yellow for β-strand, pink for α-helix, gray for random coil; (**b**) full-length 3D model of REC1 predicted de novo by AlphaFold 3 and colored by per-residue pLDDT (blue, high confidence; red, low confidence), showing the high-confidence L7/L12 carrier core and the low-confidence extended epitope arrays; (**c**) Ramachandran plot for stereochemical quality assessment of the optimal homology model. Deep green regions indicate favored conformations, light green regions indicate allowed conformations, and each dot represents the backbone dihedral angles (φ, ψ) of a single residue; (**d**) intrinsic disorder profile of REC1 predicted by AIUPred. The gray horizontal line denotes the disorder threshold of 0.5, and the distributions of PFAM conserved domains and additional annotation tracks (XP DIS, ELM, PTM) are shown at the bottom; (**e**) integrated intrinsic disorder and structural confidence profile of REC1 predicted by metapredict V3. The red curve denotes the residue-wise disorder score, and the blue curve denotes the simulated per-residue pLDDT structural confidence score (right y-axis). Red shaded areas mark the three identified intrinsically disordered regions, and the red dashed line represents the disorder threshold of 0.5.

PSIPRED-based secondary structure prediction (Figure 3a) showed that REC1 has a modular architecture that aligns well with our design rationale. The N-terminal portion, which includes the tPA signal peptide, PADRE epitope, and EAAAK linkers, is mainly α-helical, interrupted here and there by short random-coil and β-strand stretches. For the truncated L7/L12 carrier domain (residues 47–169), the prediction returned a discontinuous α-helical framework dotted with short β-strands and connecting loops; this arrangement preserves the domain’s natural propensity to fold into a globular structure.

The CTL epitope tandem array (residues 175–239), comprising five CTL epitopes joined by AAY linkers, contained α-helical segments alternating with coil regions within each epitope; the AAY linkers themselves adopted either α-helical or coil conformations. The Th epitope array (residues 248–342), in which five Th epitopes are linked by flexible GPGPG sequences, was overall dominated by random coil, although individual Th epitopes retained residual secondary-structure elements such as scattered short α-helices and β-strands. As intended, the C-terminal GGGS linker and 6×His tag were fully disordered, providing ample conformational flexibility for affinity purification.

SWISS-MODEL generated three homology models. Model 02 was selected as the best based on QMEANDisCo global score, template source, and stereochemical quality. The optimal model achieved a GMQE score of 0.20 and a QMEANDisCo global score of 0.56 ± 0.07. The low GMQE is expected for chimeric multi-epitope proteins, since no full-length homologous template exists in the PDB. The only homologous template (PDB: 4v4p.1.J, 50S ribosomal protein L7/L12) covers residues 47–169 (coverage = 0.34), corresponding to the L7/L12 carrier domain.

The full-length REC1 structure was predicted de novo with AlphaFold 3 (25 models; best ranking score 0.5963). In contrast to the earlier carrier-only homology model, the complete 357-residue chain was modeled, with a bipartite confidence distribution. The L7/L12 carrier (residues 47–169) was the only confidently folded domain (mean pLDDT 81.5; median 88.6; 81% of residues ≥ 70), whereas the CTL array (175–242; mean pLDDT 49.1) and Th array (248–347; mean pLDDT 35.8) were low-confidence and extended, consistent with the AIUPred/metapredict disorder predictions. The PADRE epitope (29–41; mean pLDDT 63.8) was intermediate and the tPA signal peptide (1–23) low-confidence, as expected. PAE analysis supported this interpretation: intra-domain PAE for the carrier was 15.3 Å, whereas the relative positioning of the CTL and Th arrays to the carrier was uncertain (28.6 and 30.9 Å) (Figure S2). As shown in Figure 3b, the model therefore displays a compact, high-confidence carrier core with an extended, low-confidence epitope region.

Geometry validation with MolProbity was favorable for the full-length AlphaFold 3 model: Ramachandran favored 87.89%; outliers 6.48%; rotamer outliers 2.61%; C-beta deviations 1; clashscore 1.99; MolProbity score 1.88. For the carrier (121 evaluable residues), the favored fraction was 83.47% with 8.26% outliers (10/121), clashscore 2.80 and score 1.82. These values are independently consistent with the carrier-only SWISS-MODEL Ramachandran assessment (81.82% favored; 6.61% outliers; Figure 3c), obtained with a different modeling and validation method. The Ramachandran outliers were localized to the flexible L7/L12 hinge (82–99), the EAAAK linker at residue 172, the disordered arrays, and the terminal tag, with no outliers in the folded helices, consistent with the pLDDT distribution.

AIUPred disorder prediction revealed a segmental pattern across the full sequence (Figure 3d). The N-terminal tPA signal peptide (residues 1–30) had high disorder scores, consistent with the inherent flexibility of secretory signal peptides. The conserved ribosomal carrier domain mostly stayed below the 0.5 threshold, indicating an overall stably folded state; a single narrow peak at residues 80–100 corresponded to a short flexible inter-helical loop within the domain. By contrast, the C-terminal multi-epitope tandem region (residues 196–357) showed a steady rise in disorder scores, with residues 280 through the C-terminus consistently scoring near 1.0 and forming a long continuous intrinsically disordered segment, consistent with the confidence distribution of the homology model.

To further validate this binary structural profile at the confidence level, we ran Metapredict-based disorder profiling and pLDDT simulation (Figure 3e). Three intrinsically disordered regions (IDRs) totaling 147 residues were identified: IDR1 (residues 1–20, mean disorder score = 0.665) covering the N-terminal tPA signal peptide; IDR2 (residues 74–103, mean disorder score = 0.768) embedded in the L7/L12 carrier domain and corresponding to a short flexible inter-helical loop; and IDR3 (residues 261–357, mean disorder score = 0.949) forming a long continuous disordered segment in the C-terminal multi-epitope region. The simulated pLDDT profile correlated inversely with disorder. The conserved N-terminal carrier domain showed high pLDDT values (mostly 80–100), supporting confident globular folding, whereas the C-terminal multi-epitope region had lower pLDDT values (40–60), indicating low confidence for stable tertiary structure. This distribution agreed well with the local confidence of the SWISS-MODEL homology model and the AIUPred disorder profile.

### 3.7. In Silico Human Immune Simulation of REC1 via C-ImmSim

To evaluate the intrinsic immunogenicity of the REC1 antigen under well-controlled idealized conditions, we performed in silico immune simulations via the C-ImmSim platform, with two independent simulation sets representing distinct HLA haplotype backgrounds: Simulation 1 and Simulation 2.

**Figure 4:**
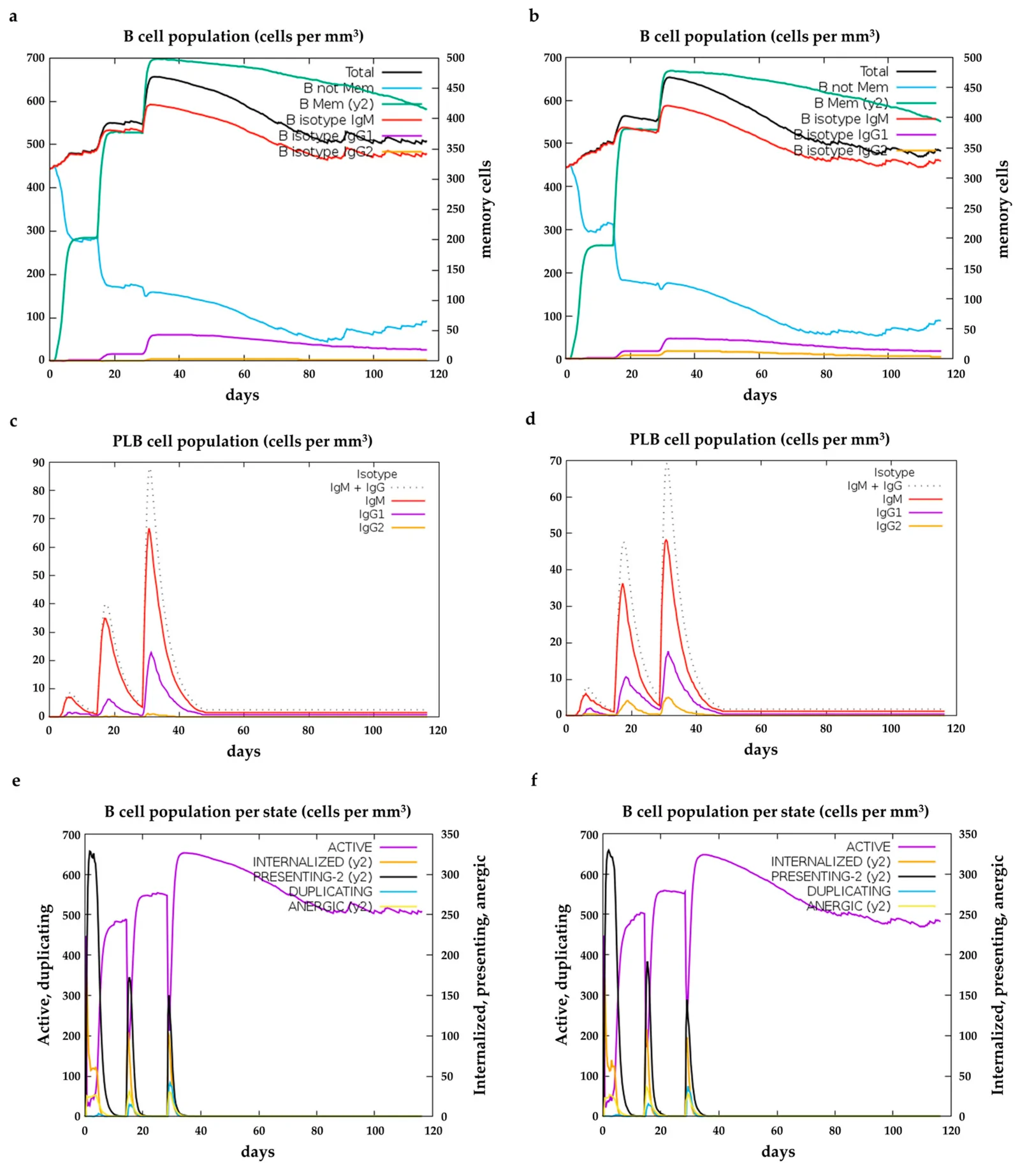
B-cell population dynamics of humoral immune response from in silico immune simulations: (**a**) Total B-cell count profile of Simulation 1 across the 120-day observation period. The x-axis indicates observation days and the y-axis indicates cell density (cells/mm^3^); (**b**) total B-cell count profile of Simulation 2 across the 120-day observation period, with identical axis settings; (**c**) PLB (plasmablast) population dynamic curve of Simulation 1, presenting effector B-cell response peaks after each immunization; (**d**) PLB population dynamic curve of Simulation 2, under the same three-dose prime-boost regimen; (**e**) activation state-resolved B-cell population counts of Simulation 1. Subsets include active, antigen-internalized, MHC II-presenting, duplicating, and anergic B-cells; (**f**) activation state-resolved B-cell population counts of Simulation 2, following the same subset classification criteria. Subpopulations labeled with the suffix (y2) are plotted against the right-hand y-axis.

Under the three-dose prime-boost regimen, both simulations predicted robust B-cell activation and plasma cell differentiation (Figure 4a–d). Total B-cell counts rose after immunization, peaking after the second dose, then declined gradually but stayed above baseline throughout the 120-day observation window. (Note that all cell counts and antibody titers are in arbitrary simulation units and cannot be directly compared with in vivo measurements.) Memory B-cell populations accumulated with each dose, peaking shortly after the second immunization and remaining elevated thereafter, suggesting effective long-term humoral memory. Plasmablast (PLB) populations showed distinct response peaks after each immunization (Figure 4c,d), with IgM-producing PLBs dominating at every peak. IgG1^−^ secreting (and, to a lesser extent, IgG2^−^ secreting) subsets expanded more strongly after each booster, and the total PLB response grew progressively across the three doses, consistent with amplification of the antigen-specific memory B-cell pool. The marked expansion of IgG-secreting subsets after boosting confirmed antibody class switching.

Activation state-resolved analysis clarified B-cell population dynamics during the response (Figure 4e,f). Upon activation, antigen-specific B-cells undergo clonal expansion, so the active, proliferating B-cell subset expanded rapidly and formed a peak that coincided with the PLB response. Antigen stimulation also caused a transient rise in B-cells that internalize antigen and present MHC class II molecules, corresponding to the processing-and-presentation phase that bridges innate recognition and T-cell-dependent B-cell activation. As antigen was cleared and the response subsided, these active and proliferating subsets declined. Non-responsive B-cells, by contrast, remained at a low, stable level throughout. B-cell state-transition trajectories were highly consistent between the two simulations, confirming stable activation and differentiation.

**Figure 5:**
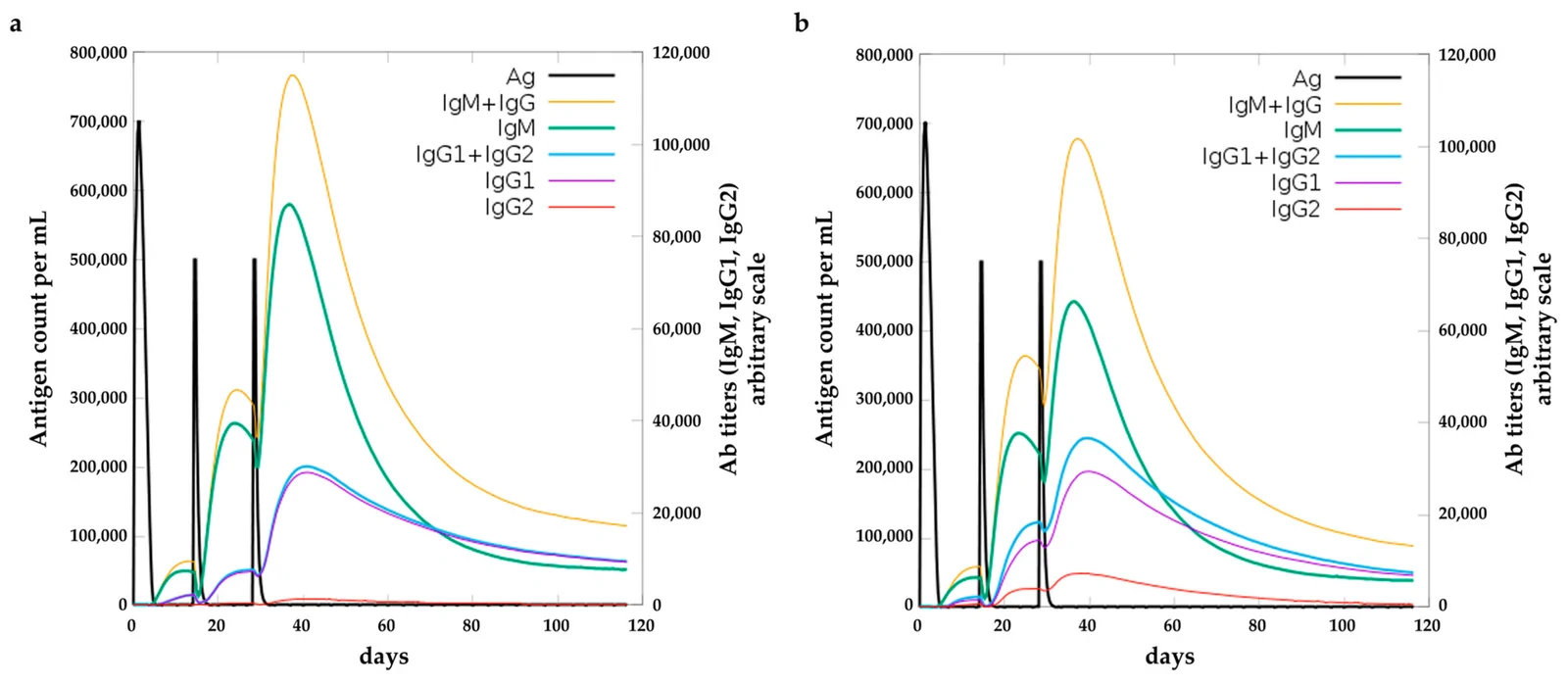
Dynamics of immunoglobulins and antigen clearance of humoral immunity at the molecular level: (**a**) Quantitative profiles of antigen, immunoglobulins and immunocomplexes of Simulation 1. Curves reflect antigen load changes and total antibody titer elevation after each immunization; (**b**) quantitative profiles of antigen, immunoglobulins and immunocomplexes of Simulation 2, with consistent detection indicators and observation window.

By the third immunization, the total antibody titre (IgM + IgG) had climbed to roughly 100,000–120,000 arbitrary units, a clear indication that each booster dose amplifies the response (Figure 5a,b). We also observed rapid antigen clearance shortly after every injection in both simulations, which is what one would expect if the vaccine-induced antibodies possess functional neutralizing and phagocytic activity.

**Figure 6:**
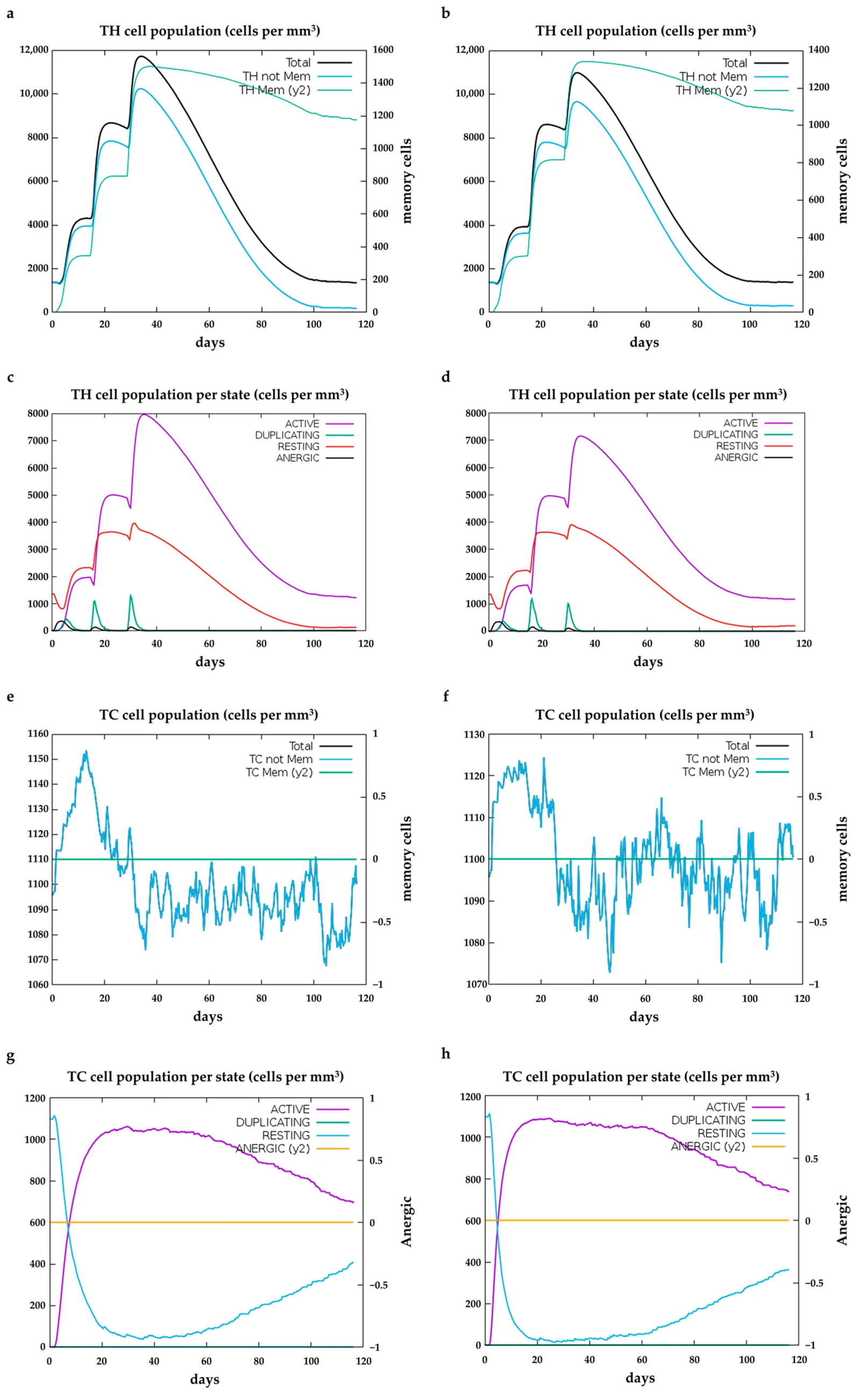
Th-cells and CTL-cells dynamics of cellular immune response: (**a**) Th-cell count profile of Simulation 1. The y-axis indicates cell density (cells/mm^3^); (**b**) total Th-cell count profile of Simulation 2, with identical axis and observation settings; (**c**) activation state-resolved Th-cell population counts of Simulation 1, reflecting Th cell polarization and memory formation; (**d**) activation state-resolved Th-cell population counts of Simulation 2, with the same subset classification rules; (**e**) total CTL-cell count profile of Simulation 1, showing moderate CTL response magnitude; (**f**) total CTL-cell count profile of Simulation 2; (**g**) activation state-resolved CTL-cell population counts of Simulation 1; (**h**) activation state-resolved CTL-cell population counts of Simulation 2, with consistent state annotation standards. Subpopulations labeled with the suffix (y2) are plotted against the right-hand y-axis.

The simulations also pointed to a strong Th-cell response. Total Th-cell numbers peaked around the third dose at approximately 11,000–12,000 cells/mm^3^ (Figure 6a,b). Memory Th-cells built up gradually following the last booster and then plateaued, suggesting that durable memory-cell-mediated immunity is achievable. Looking at the acute phase more closely, activated Th-cells were the dominant subset; resting Th-cells were the next most abundant and slowly decreased after their peak, whereas proliferative Th-cells produced only a brief, early spike (Figure 6c,d).

CTL responses were clearly detectable in both simulations, and despite different HLA backgrounds, the overall magnitudes were comparable (Figure 6e,f). Simulation 1 (HLA-B*15:01/HLA-B*54:01) and Simulation 2 (harboring the HLA-B*15:02/HLA-B*58:01 haplotype) both mounted CTL responses of similar magnitude, with total counts reaching approximately 1100–1150 cells/mm^3^. Thus, differences in predicted epitope breadth did not translate into substantially higher total CTL counts, suggesting that population size alone may not fully capture HLA-driven differences in immune breadth.

Because both simulations shared the HLA-A*01:01 haplotype, HLA-A*01:01-restricted epitopes provided a conserved basal backbone for CTL activation. Even so, the overall CTL magnitude was strongly influenced by the paired HLA-B haplotype. Activation state-resolved CTL analysis (Figure 6g,h) showed consistent patterns across the two simulations: active T-cells expanded and declined in parallel with total CTL numbers, duplicating subsets showed rapid but brief early peaks, and anergic T-cells remained at stable low baseline levels throughout.

**Figure 7:**
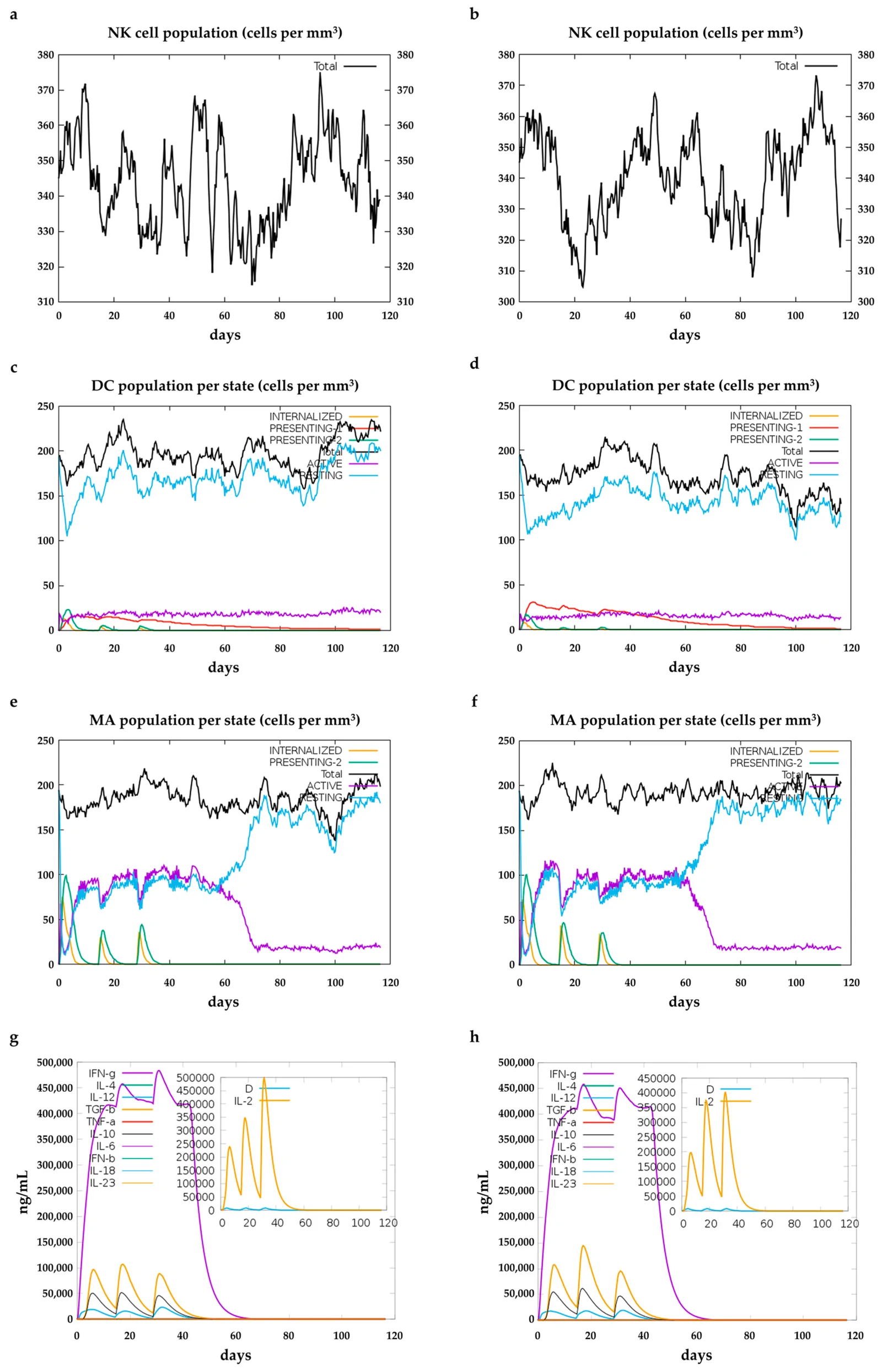
Innate immune cell dynamics and cytokine expression profiles: (**a**) Total NK (natural killer) cell count curve of Simulation 1, reflecting innate immune activation upon antigen stimulation; (**b**) total NK cell count curve of Simulation 2, with consistent observation settings; (**c**) activation state-stratified DC (dendritic cell) population counts of Simulation 1; (**d**) activation state-stratified DCs population counts of Simulation 2, with the same subset classification; (**e**) activation state-stratified MA (macrophage) population counts of Simulation 1; (**f**) activation state-stratified MAs population counts of Simulation 2, with consistent state annotation; (**g**) concentration profiles of cytokines and interleukins of Simulation 1, including key Th1-type effector molecules IFN-γ and IL-2; (**h**) concentration profiles of cytokines and interleukins of Simulation 2, covering the same panel of inflammatory and immunoregulatory factors.

Innate immune cell and cytokine dynamics are summarized in Figure 7. Total NK-cell counts fluctuated after each antigen challenge, reflecting rapid innate activation (Figure 7a,b). Dendritic cells (DCs) remained mostly resting, with minimal activation and antigen presentation throughout (Figure 7c,d). Macrophages (MAs), by contrast, underwent rapid state transitions after each immunization, with transient spikes in the internalized and presenting phenotypes. During the first 60 days, the activated MA population was slightly higher than or comparable to the resting population; after day 60, however, activated MAs declined sharply while resting MAs surged and became the dominant population for the remainder of the observation period (Figure 7e,f).

Cytokine profiles showed a clear Th1 bias in both simulations (Figure 7g,h). After each vaccination, Th1-type effector molecules such as IFN-γ and IL-2 were markedly upregulated, peaking in parallel with the T-cell response, a favorable profile for vaccines against intracellular pathogens like *Brucella*. Pro-inflammatory and immunomodulatory cytokines also rose after vaccination, together forming an innate-adaptive activation cascade that drives the onset and progressive amplification of the adaptive response.

The two simulation groups produced highly consistent results: B-cell response patterns, peak antibody titres, Th-cell response intensity, and the core Th1-biased polarization pattern were essentially identical. This is not surprising, as both groups shared the HLA-A*01:01 haplotype and HLA-DRB1 background, giving them similar baseline immune activation.

Because of platform input limits, only HLA-DRB1-restricted Th epitopes (REC1-T1, REC1-T2, REC1-T4) could be included; the DQ-restricted REC1-T3 and DP-restricted REC1-T5 were not modeled. The simulated Th responses are therefore a partial estimate and likely understate REC1’s full helper T-cell activation potential. Overall, the concordance of core protective features, robust humoral immunity and Th1-polarized cellular immunity, across different HLA-B backgrounds indicates that REC1 has stable intrinsic immunogenicity. CTL response amplitude, however, varies with individual HLA class I backgrounds, underscoring the importance of HLA diversity for vaccine-induced cytotoxic T-cell immunity.

### 3.8. Molecular Docking, Interface Analysis and Peptide-MHC Modeling

**Figure 8:**
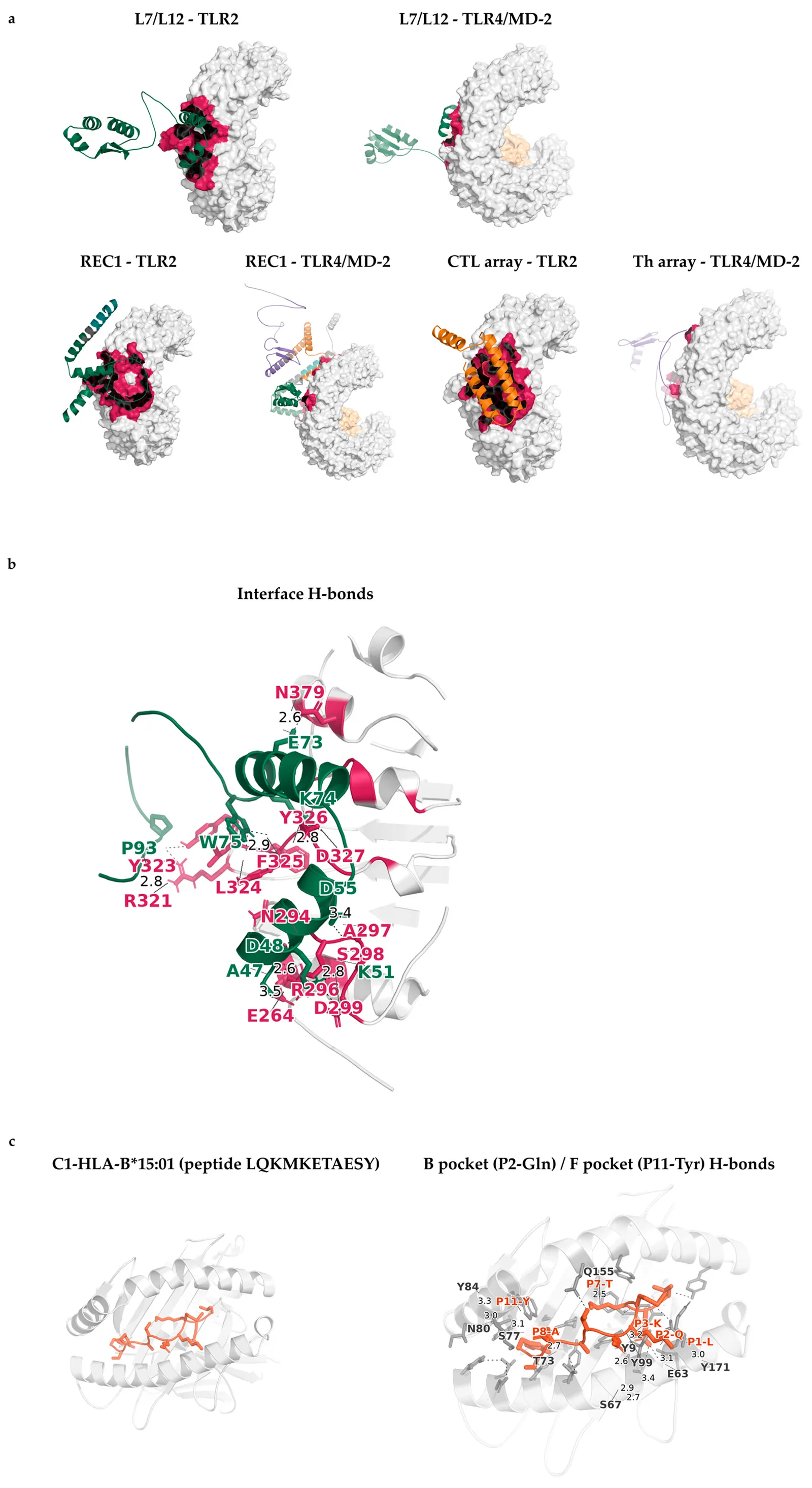
Molecular docking and representative peptide–MHC modeling of REC1: (**a**) Overview of the best pocket-directed HADDOCK complexes: L7/L12-TLR2, L7/L12-TLR4/MD-2, full-length REC1-TLR2, REC1-TLR4/MD-2, CTL array-TLR2, and Th array-TLR4/MD-2. Receptors are shown as a white surface, and ligand regions are colored by module; the predicted interface is highlighted in magenta; (**b**) close-up of the L7/L12-TLR2 interface, with predicted hydrogen bonds (dashed) and contacting residues labeled; (**c**) representative peptide–MHC model of C1 (LQKMKETAESY) bound to HLA-B*15:01 (left, peptide in orange within the groove; right, B and F pocket hydrogen bonds). Carrier interfaces with a low-confidence fraction <0.20 (TLR2, CD14, C1q, DC-SIGN) are treated as well-defined; the L7/L12-TLR4/MD-2 and full-length/array interfaces are hypothesis-generating. Full metrics are given in Table S10; extended sensors and source antigen controls are shown in Figures S3 and S4.

To examine how REC1 might engage innate receptors at the atomic level, we docked the full-length construct, its individual modules, and the five source antigens against TLR2 and TLR4/MD-2, and included an extended sensor panel (CD14, C1q, DC-SIGN) (Figure 8a; Table S10). The L7/L12 carrier formed well-defined interfaces (low-confidence interface fraction < 0.20) with TLR2 (BSA 1025 Å^2^; 17 predicted hydrogen bonds; one salt bridge; six hydrophobic and two π contacts; low-confidence fraction 0.105) (Figure 8b), CD14 (798 Å^2^; 18 hydrogen bonds; four salt bridges; nine hydrophobic contacts; 0.00), C1q (536 Å^2^; nine hydrogen bonds; three salt bridges; 0.00) and DC-SIGN (712 Å^2^; 18 hydrogen bonds; four salt bridges; 0.00) (Figure S3). The pocket-directed L7/L12-TLR4/MD-2 interface was larger (1324 Å^2^; 23 hydrogen bonds; five salt bridges) but its low-confidence fraction was 0.29, above the 0.20 gate, and is therefore reported separately rather than grouped with the well-defined interfaces.

Interfaces involving the full-length REC1 construct were larger in buried surface but were dominated by the disordered epitope arrays and are treated as hypothesis-generating. Full-length REC1-TLR2 had a BSA of 1189 Å^2^ (26 hydrogen bonds) but a low-confidence fraction of 0.50, and full-length REC1-TLR4/MD-2 had a BSA of 1654 Å^2^ (22 hydrogen bonds; 23 hydrophobic contacts) with a low-confidence fraction of 0.48. The isolated CTL (0.56) and Th (1.00) arrays likewise produced only hypothetical contacts. In the unrestrained mode, contacts with receptor ligand-site residues were observed in 9 of 17 complexes, but interface residue overlap between the two modes was low (Jaccard 0–0.37), and the unrestrained criterion is lenient, so pocket engagement is presented as a targeted-docking hypothesis rather than an independently validated binding mode.

Among the individual source antigen controls, DnaK (low-confidence fraction 0.11), Omp25 (0.00), Omp31 (0.00) and BamA (0.00) formed well-defined interfaces with TLR2 (BSA 778-1460 Å^2^; 8-16 hydrogen bonds), whereas Omp19 (0.32) was above the gate (Figure S4). The modular arrays did not enlarge the well-defined interface beyond that of the carrier or individual antigens; the additional buried surface came from disordered regions. Collectively, the L7/L12 carrier provides the structurally well-defined component of predicted receptor engagement, while the epitope arrays remain the antigenic but conformationally flexible component.

Finally, the representative peptide–MHC model of C1 bound to HLA-B*15:01 showed the canonical binding geometry: the P2-Gln and PΩ-Tyr anchors were positioned in the B and F pockets (anchor backbone RMSD 0.458 Å versus the template), the peptide lay fully within the binding groove with 12 peptide–MHC hydrogen bonds and no steric clashes (Figure 8c). This provides a structural rationale for T-cell recognition of the C1 epitope by its restricting allele. Because TCR recognition is clonotype-specific and no matched TCR sequence or template was available, pMHC-TCR docking is deferred to future work. All docking and modeling results are predicted potential contacts rather than binding affinities and require experimental confirmation, such as TLR-blocking assays or surface plasmon resonance.

The overall docking profiles, representative receptor-antigen interaction interface, and peptide–MHC conformation are summarized in Figure 8.

## 4. Discussion

Multi-epitope subunit vaccines offer a safer alternative to live attenuated vaccines for brucellosis control, but their clinical translation has been slow, largely because in vivo epitope validation is scarce, and few designs are optimized for the HLA profile of the target population [69,70,71]. To address this, we built an integrated reverse vaccinology workflow that combines bioinformatic prediction with experimental immunogenicity confirmation, and developed REC1, a multi-epitope candidate optimized for the Chinese population.

Choosing the right carrier backbone is a key design decision for modular subunit vaccines. Although carrier-derived T-cell epitopes can boost responses to the fused antigen, the carrier also affects expression yield and stability of the recombinant fusion protein. For intracellular pathogens such as *Brucella*, a carrier with intrinsic Th1-polarizing activity is preferable to maximize cellular immune protection.

For intracellular pathogen vaccines, the carrier must support soluble expression while amplifying the Th1 polarization needed for *Brucella* clearance. We selected L7/L12 over Omp28 and BLS chiefly for its well-characterized immunomodulatory activity: it promotes dendritic cell maturation and IL-12 secretion, driving naïve Th-cell toward a Th1 effector phenotype. Because protection against intracellular *Brucella* depends almost entirely on IFN-γ-mediated macrophage activation, this endogenous adjuvant effect is particularly well suited to *Brucella* vaccine design. Omp28, by contrast, functions mainly as an immunodominant antigen that elicits mixed humoral and cellular responses but has no standalone Th1-adjuvant activity. BLS serves primarily as a multimeric antigen-display platform through its decameric structure, with weak intrinsic immunostimulatory activity and little capacity for Th1 polarization. These functional differences point to truncated L7/L12 as the preferred carrier, since it aligns most closely with the Th1-biased immunity needed for protection against *Brucella*.

Antigens were selected for functional complementarity and documented roles in *Brucella* pathogenesis, drawing from two distinct groups. The first comprises outer membrane proteins (Omp19, Omp25, Omp31), which mediate host cell adhesion, intramacrophage survival, and immune evasion and are well-established targets for combined humoral and cellular immunity [72,73,74]. A 10-25 kb genomic deletion spanning the *Omp31* gene in *B. abortus* accounts for its natural absence in this species; Omp31 is thus present in all major pathogenic *Brucella* species except *B. abortus* and has been used as a species-differentiation marker. This species-specific loss does not diminish Omp31’s value as a vaccine target, since it remains highly conserved and immunogenic across the other principal zoonotic *Brucella* species, and it also offers a theoretical basis for developing DIVA-compatible diagnostics in future work.

The second group consists of intracellular stress-response and envelope-assembly factors, represented by DnaK and BamA. Both support bacterial survival under intracellular stress and maintain cell-envelope integrity, and elicit strong T-cell responses during natural infection [75,76,77]. GroEL was excluded because of suboptimal in vivo immunogenicity and functional redundancy with DnaK.

All selected antigens and, importantly, their embedded REC1 epitopes, are conserved across major pathogenic *Brucella* species, providing epitope-level support for potential broad-spectrum protection [78,79,80,81]. Although Omp31 is absent from *B. abortus* at the gene level, an epitope-level analysis confirmed that all ten non-Omp31 epitopes, including their predicted MHC anchor and TCR-facing residues, are 100% conserved, providing epitope-level rather than solely whole-protein coverage of this species.

We used a hierarchical linker strategy to minimize junctional neoepitopes and preserve independent domain function, an approach that differs from many single-linker multi-epitope constructs [82]. AAY motifs were used exclusively for CTL epitope ligation to ensure precise proteasomal cleavage at each epitope C-terminus, while GPGPG sequences separated Th epitopes to facilitate lysosomal processing and MHC-II loading [83]. Rigid EAAAK spacers isolated the carrier domain from the epitope arrays to avoid steric disruption of L7/L12 folding, and a flexible GGGS linker separated the His-tag from the antigenic region. This tiered design reduces non-native flanking sequences that could compete with authentic epitopes for MHC binding, a common drawback of simple tandem epitope constructs.

The discrepant allergenicity predictions reflect differences in algorithm design. AlgPred 2.0 and AllergenFP v1.1 base their calls on global physicochemical features rather than homology to confirmed allergens or IgE epitopes, and are known to produce high false-positive rates for Ala/Gly-rich proteins. Under FAO/WHO guidelines, which prioritize sequence homology and known IgE epitope matches over physicochemical similarity, the uniformly negative results from all sequence-based analyses support a low allergenic risk for REC1. Confirmatory experiments, including serum IgE binding assays, will be needed to fully exclude allergenic potential.

ToxinPred 2.0 likewise classified REC1 as non-toxic, consistent with the allergenicity results and further supporting a favorable preliminary safety profile. The absence of detectable toxic motifs and of significant homology to known toxic proteins suggests that REC1 lacks the primary molecular features associated with protein toxicity. In silico prediction cannot fully replace in vitro cytotoxicity assays or in vivo safety evaluations, but together with the low-allergenicity evidence, these results provide convergent computational support for a low theoretical safety risk and justify further experimental validation in later development stages.

A defining feature of REC1 is its bipartite architecture: a stably folded L7/L12 carrier core coupled to an extended, intrinsically disordered multi-epitope tail at the C-terminus. Most conventional globular subunit vaccines do not have this arrangement: in a conventional globular antigen, the epitopes tend to be buried within the folded protein and are therefore less accessible to the antigen processing machinery. The disordered tail in REC1 avoids steric clashes between neighboring epitopes and gives proteasomal and lysosomal proteases better access, which should promote efficient epitope release and MHC loading. There is also evidence that unstructured antigen regions are taken up more readily by antigen-presenting cells, potentially adding another layer of cellular immune amplification.

The tPA signal peptide, PADRE pan-DR epitope, and L7/L12 carrier were included to complement epitope-driven immunity: the signal peptide enhances antigen uptake and processing, PADRE broadens HLA-II coverage across populations, and the L7/L12 backbone supplies endogenous Th1-polarizing activity. This combinatorial design is intended to offset the relatively weak immunogenicity typical of short synthetic epitope arrays.

To place REC1 in the context of published *Brucella* multi-epitope constructs, we compared its predicted physicochemical profile with three representative in silico designs. REC1 (357 residues; 35.86 kDa; pI 5.64; instability index 25.25; aliphatic index 79.89; GRAVY −0.015) is markedly smaller than the 806-residue, 86.25 kDa construct reported by Li et al., while retaining a comparably low instability index (20.79), indicating that REC1 achieves favorable predicted stability without a large fusion mass [69]. The construct of Jalal et al. (~33 kDa; pI 5.03; instability index 26.98; GRAVY −0.160) is close to REC1 in size and predicted stability [79]. The Gharazi et al. construct (387 residues; 38.94 kDa; pI 6.16; GRAVY −0.641) was reported with an instability index of 52.29, which exceeds the conventional stability threshold of 40, whereas REC1’s lower index (25.25) is comparatively favorable [84]. In common with these earlier constructs, REC1 combines an ordered carrier domain with flexible linker-joined epitope arrays; its distinguishing features are the population-informed HLA selection and the experimental pre-screening of each peptide before assembly. Its near-neutral GRAVY and high aliphatic index predict a soluble, readily expressed protein suitable for recombinant production in *E. coli*.

C-ImmSim simulations provide a preliminary mechanistic picture of REC1 immunogenicity under controlled conditions. With a three-dose prime-boost schedule, the simulations predicted robust and sustained humoral immunity, including rising antibody titres, isotype switching, and persistent memory B-cell populations. At the cellular level, the vaccine elicited strong Th-cell responses with a pronounced Th1 polarization profile, evidenced by high IFN-γ and IL-2 levels, which is particularly desirable for protection against intracellular pathogens like *Brucella*. The consistency of core immune features across different HLA-B backgrounds supports REC1’s stable intrinsic immunogenicity. Because REC1 was designed as a T-cell-focused candidate and does not include explicitly predicted B-cell epitopes, humoral responses are expected to be largely Th-cell-dependent.

C-ImmSim makes several simplifying assumptions that limit direct extrapolation of its outputs to real in vivo outcomes. Most importantly, the model does not include LPS, a major innate immune trigger that shapes the Th1-polarizing microenvironment during natural infection. The Th1 bias observed in the simulation therefore reflects the intrinsic properties of the REC1 protein alone, not the full adjuvant effects achievable with a complete vaccine formulation.

In addition, the platform simulates only a simplified immune-cell network and does not capture tissue-level immune trafficking or mucosal immunity, both central to *Brucella* host interactions during natural exposure. For these reasons, simulation outputs should be interpreted as qualitative trends rather than reliable predictions of the actual magnitude of in vivo immune responses.

The ≥3% carrier frequency cutoff used to construct the HLA panel was an operational threshold rather than a universal standard, and we therefore validated it empirically instead of invoking convention. The cumulative carrier coverage analysis places the chosen cutoff on a clear saturation plateau: for HLA class I, 27.5% of the observed functional alleles (49/178) captured 99.90% of cumulative carrier coverage, and the remaining 129 low-frequency alleles added only 0.05 pp, while the HLA-DRB1, -DQA1 and -DQB1 panels were similarly saturated. Two qualifications should be noted. First, because each allele was assigned the highest carrier frequency reported across Chinese subpopulations, the curves are descriptive and may slightly overestimate coverage in any single population. Second, the HLA-DPB1 panel retained a somewhat larger long tail gain (+1.45 pp), and single-chain DQA1/DQB1/DPB1 frequencies do not represent functional α/β heterodimers; these screening-level simplifications are addressed at the epitope level by separate functional heterodimer and IEDB population coverage analyses, in which the final REC1 set attained 98.88% combined coverage in the China Oriental population. The 3% cutoff therefore controls screening throughput without materially compromising the population coverage of the final, experimentally immunoreactive epitope set.

The molecular docking and peptide–MHC analyses provide a structural basis for REC1 receptor engagement and presentation. The L7/L12 carrier, previously established as a self-adjuvanting Th1 driver, formed well-defined predicted interfaces with TLR2, CD14, C1q, and DC-SIGN, supporting the design rationale that the carrier, not the disordered epitope arrays, mediates the structured component of innate engagement. The full-length and array interfaces, although larger, were dominated by low-confidence/disordered regions and are treated as hypotheses, consistent with the deliberately ordered-carrier/disordered-epitope architecture, in which the arrays are intended to remain accessible for processing rather than to fold. The representative C1-HLA-B*15:01 model confirms the epitope can be accommodated in the canonical groove geometry. We note two boundary conditions: the CD14 structure used is murine, and DC-SIGN recognizes glycans, whereas recombinant REC1 produced in *E. coli* is not glycosylated. Docking scores are not binding affinities, and the predicted interfaces should be tested experimentally (e.g., TLR-blocking antibodies, HEK-Blue reporter cells, or SPR/BLI).

Finally, because the well-defined interface maps to the ordered carrier rather than the epitope arrays, interface-guided optimization of EAAAK linker spacing and targeted point mutations, followed by re-docking and experimental binding measurements, is a rational next step. Such design iterations must be validated experimentally, since docking scores rank poses but do not quantify binding affinity.

Several limitations should be borne in mind. First, ELISpot validation used only 4–5 animals per group and should therefore be regarded as an exploratory, underpowered screen. Responder proportions have very wide exact 95% confidence intervals. With these group sizes and the number of comparisons, exact two-sided Mann–Whitney tests could not attain Bonferroni-adjusted significance even at complete group separation; thus, group-level statistics are descriptive rather than confirmatory. Although the individual positivity rule and ≥50% responder threshold were pre-specified, the decision to use responder rate as the primary selection criterion was made after observing that adjusted *p* values were uninformative in this small, discrete, low-background setting. This cutoff is a pragmatic prioritization rule rather than a multiplicity-adjusted test of immunodominance; it may favor broadly reactive peptides over the most potent sequences [84,85,86,87,88,89], and it requires independent validation in a larger cohort with a pre-specified analysis plan. Second, epitopes were predicted against human HLA alleles, but in vivo immunogenicity was assessed only in BALB/c (H-2^d^) mice, whose peptide-binding motifs differ from those of human HLA [90,91]. The murine data support natural processing and presentation of the selected sequences in an H-2^d^ context, but they do not establish HLA-restricted immunodominance or human immunogenicity. BALB/c was also the sole animal model and has a relative Th2 propensity, which may limit prediction of human Th1 responses. Future validation should include HLA-transgenic mice and/or human DC-T-cell or PBMC assays from ethnically diverse donors. Third, the HLA frequencies used for population coverage modeling were Chinese-specific rather than global. Because *Brucella* infection is distributed worldwide, cross-ethnic population coverage and vaccine performance remain unknown. The class II estimates also involve explicit approximations: the IEDB calculator models single chains rather than paired heterodimers; HLA-DP coverage was approximated at the DPB1 level with a fixed DPA1*01:03 α-chain; and HLA-DQ coverage relied on relatively small Xinjiang cohorts under a Hardy–Weinberg assumption and was therefore reported separately. These estimates should be recalculated using global, ethnically diverse datasets and paired-haplotype information, with uncertainty intervals. Fourth, infection was established with the attenuated BP26-deletion strain M5-90Δ26 rather than virulent field isolates. Attenuation may alter intracellular persistence, antigen load, antigen processing, and innate activation, so responses to this strain may not fully reproduce those induced by virulent *Brucella*. Although the present candidate set does not include BP26-derived epitopes, the BP26 deletion also limits future extension of the platform: M5-90Δ26 cannot validate BP26-containing constructs or fully represent the antigen repertoire expressed by the BP26-intact parental strain, and such candidates would require a BP26-expressing strain or virulent isolate. Finally, no challenge or protection assay was performed. Immunogenicity, structural characterization and theoretical HLA coverage do not establish protective efficacy. Future confirmatory studies should use virulent isolates in appropriate biosafety facilities and assess bacterial burden in the spleen and liver, histopathology, survival, Th1 and memory responses, safety, and efficacy relative to an established reference vaccine.

Compared with most published brucellosis multi-epitope vaccines, which rest exclusively on computational prediction, our design has two strengths that support translational potential: in vivo immunogenicity screening of both MHC-I and MHC-II peptides in a chronic infection model, and optimization against high-frequency HLA alleles from the target Chinese population. Critically, coverage analysis of the final incorporated epitopes predicted that 91.17% of the China Oriental population would recognize at least one REC1 T-cell epitope on the conservative class I + DR estimate, rising to 98.88% when HLA-DP restrictions were included, while the HLA-DQ component alone was estimated at 57.75–79.04% (68.17% in Han) under strict four-digit haplotype counting. This quantifies the coverage delivered by the final construct and distinguishes it from the allele frequencies used only at the screening stage. Murine responses only support peptide immunoreactivity during genuine infection; validation in HLA-transgenic mice or human PBMCs will be needed to establish under human HLA restriction.

As a non-replicating subunit candidate, REC1 avoids the residual virulence and abortion risks associated with live attenuated veterinary vaccines and the 104M candidate strain. It may also enable serological differentiation between vaccinated and naturally infected hosts, since it carries no predicted risk of residual virulence, abortion, or occupational infection.

Looking ahead, the immediate priority is HLA-matched immunogenicity validation; we plan to use HLA-transgenic mice as well as PBMCs from individuals with known *Brucella* exposure. Once REC1 has been recombinantly expressed and purified, we will conduct in vivo challenge experiments to determine whether it confers protection. In parallel, we intend to optimize Th1-biased adjuvant formulations and delivery platforms, both of which will be important for moving the candidate toward clinical translation.

## 5. Conclusions

By integrating in silico prediction with in vivo epitope validation in a reverse vaccinology pipeline, we developed and comprehensively characterized REC1, a multi-epitope brucellosis vaccine candidate optimized for high-frequency HLA alleles in the Chinese population. A frequency-ranked saturation analysis confirmed that this high-frequency panel captured >99% of cumulative HLA class I carrier coverage and was at or near saturation for HLA-DRB1, -DQA1, and -DQB1, indicating that the 3% screening threshold did not materially restrict population representation. Population-coverage modeling further predicted that the final epitopes would be recognized by more than 91% (and up to 98.88% when HLA-DP was included) of the China Oriental population, supporting the population breadth of the design. All embedded peptides showed IFN-γ immunogenicity in a murine chronic infection model, were designated candidate immunoreactive epitopes, and were 100% conserved at the epitope level across major *Brucella* species, with the two Omp31 epitopes absent only from the Omp31-deleted *B. abortus*. In silico analyses further revealed favorable physicochemical properties, a rationally designed architecture featuring an ordered carrier domain and a disordered epitope region, and the simulated potential to induce Th1-polarized humoral and cellular immunity with long-term memory. Validation in HLA-transgenic models and challenge studies will be needed to establish clinical translational value. Atomic-level docking further predicted that the L7/L12 carrier forms well-defined interfaces with TLR2, CD14, C1q, and DC-SIGN, while a representative peptide–MHC model confirmed canonical groove binding, providing a structural basis for innate engagement and T-cell presentation.

This study establishes a methodologically validated pipeline for brucellosis multi-epitope vaccine design, and REC1 represents a promising early candidate for downstream efficacy testing.

## Figures and Tables

**Table 1:** UniProt accession numbers of the six candidate proteins.

Candidate Proteins	Accession
GroEL	Q8YB53
DnaK	Q8YE76
Omp19	P0A3P1
Omp31	P0A3U4
Omp25	Q8YEP8
BamA	Q8YHH0

**Table 2:** Summary of MHC-I and MHC-II epitope predictions for the six candidate antigens.

Antigen	MHC-I				MHC-II			
	Total	Unique ^1^	SB ^2^	No. of SB-Associated HLA Alleles	Total	Unique ^1^	SB ^3^	No. of SB-Associated HLA Alleles
GroEL	131,075	2675	124	34	32,984	532	221	62
DnaK	153,370	3130	164	46	38,626	623	278	62
Omp19	40,670	830	34	32	10,106	163	41	30
Omp31	56,105	1145	59	37	14,012	226	62	37
Omp25	53,655	1095	66	35	13,392	216	80	53
BamA	188,650	3850	241	47	47,554	767	358	62
Total	623,525	12,725	688	48	156,674	2527	1040	62

^1^ The counts after removing duplicate sequences. ^2^ Strong binder counts, defined as peptides with a %Rank ≤ 0.5 (NetMHCpan 4.1). ^3^ Strong binder counts, defined as peptides with a %Rank ≤ 2 (NetMHCIIpan 4.1).

**Table 3:** Synthetic MHC-I and MHC-II peptides used for IFN-γ ELISpot validation (all with %Rank = 0.01).

Peptide No.	Sequence	Source Antigen	Top Allele
1	QQIEETTSDY	GroEL	HLA-B*15:01
2	LQKMKETAESY	DnaK	HLA-B*15:01
3	FPNAPSTDMSA	Omp19	HLA-B*54:01
4	TAAPVDTFSW	Omp31	HLA-B*58:01
5	MVYGTGGLAY	Omp31	HLA-B*15:02
6	MPYIAGGVAF	Omp25	HLA-B*35:01
7	ATDNVLLRLEY	Omp25	HLA-A*01:01
8	RASVGVSLMW	BamA	HLA-B*58:01
9	SSNAVLDPSTNEY	BamA	HLA-A*01:01
10	ITDNFSAGIAY	BamA	HLA-A*01:01
11	QADEETLRRFY	BamA	HLA-A*01:01
12	YSVDQGPRAY	BamA	HLA-A*01:01
13	RYWMGGTTYF	BamA	HLA-A*24:02
14	SISKENTTIVDGAGQ	GroEL	HLA-DQA1*01:03/DQB1*06:03
15	QAMYEAAQAAEGAGA	DnaK	HLA-DRB1*10:01
16	KQLVLYDANGGTVAS	Omp19	HLA-DRB1*15:01
17	TGGYIGINAGYAGGK	Omp31	HLA-DRB1*10:01
18	TSTSAYAADAIVAQE	Omp25	HLA-DQA1*01:02/DQB1*06:09
19	KTTFKGNYYQTLSQE	BamA	HLA-DPA1*01:03/DPB1*04:02

**Table 4:** IFN-γ ELISpot responses to individual MHC-I and MHC-II peptides in *Brucella*-infected mice.

Peptide	Responders, n/N (Responder Rate, %)	Net SFU/10^6^, Mean	Unadjusted Exact *p*	Bonferroni-Adjusted *p*	Responder 95% CI	Candidate (Yes/-)
1 (GroEL)	2/5 (40%)	45	0.0079	0.1032	0.0527–0.8534	-
2 (DnaK)	3/5 (60%)	54	0.0079	0.1032	0.1466–0.9473	Yes
3 (Omp19)	3/5 (60%)	58	0.0079	0.1032	0.1466–0.9473	Yes
4 (Omp31)	2/5 (40%)	51	0.0079	0.1032	0.0527–0.8534	-
5 (Omp31)	4/5 (80%)	68	0.0079	0.1032	0.2836–0.9949	Yes
6 (Omp25)	2/5 (40%)	67	0.0079	0.1032	0.0527–0.8534	-
7 (Omp25)	4/5 (80%)	83	0.0079	0.1032	0.2836–0.9949	Yes
8 (BamA)	3/5 (60%)	58	0.0079	0.1032	0.1466–0.9473	Yes
9 (BamA)	3/5 (60%)	58	0.0079	0.1032	0.1466–0.9473	Yes
10 (BamA)	1/5 (20%)	39	0.0079	0.1032	0.0051–0.7164	-
11 (BamA)	2/5 (40%)	61	0.0317	0.4127	0.0527–0.8534	-
12 (BamA)	4/5 (80%)	60	0.0079	0.1032	0.2836–0.9949	Yes
13 (BamA)	1/5 (20%)	44	0.0397	0.5159	0.0051–0.7164	-
14 (GroEL)	1/4 (25%)	28.75	0.0286	0.1714	0.0063–0.8059	-
15 (DnaK)	4/4 (100%)	66.25	0.0286	0.1714	0.3976–1.0000	Yes
16 (Omp19)	3/4 (75%)	66.25	0.0286	0.1714	0.1941–0.9937	Yes
17 (Omp31)	4/4 (100%)	147.5	0.0286	0.1714	0.3976–1.0000	Yes
18 (Omp25)	3/4 (75%)	90	0.0286	0.1714	0.1941–0.9937	Yes
19 (BamA)	4/4 (100%)	70	0.0286	0.1714	0.3976–1.0000	Yes

Peptide comparisons used exact two-sided Mann–Whitney tests with Bonferroni correction within the 13-test MHC-I and 6-test MHC-II families; no comparison reached adjusted *p* < 0.05. Responder proportions are shown with exact Clopper-Pearson 95% CI.

**Table 5:** Predicted physicochemical parameters and antigenicity of candidate protein carrier.

Physicochemical Parameters	L7/L12	Outer Membrane Protein Omp28 (BP26)	Lumazine Synthase BLS
Number of amino acids	124	250	157
Theoretical isoelectric point (*pI*)	4.79	6.39	4.45
Molecular weight (Da)	12.55	26.55	16.81
Instability index (II)	18.55	27.93	31.80
Aliphatic index	108.87	92.16	93.38
Grand average of hydropathicity (GRAVY)	0.119	−0.030	−0.013
VaxiJen antigenicity score	0.6244	0.5381	0.4891

**Table 6:** MHC-I and MHC-II peptides used to construct REC1.

Epitope ID	Sequence	Source Antigen	Top Allele	Type	SB Allele Count
REC1-C1	LQKMKETAESY	DnaK	HLA-B*15:01	CTL (MHC-I)	3
REC1-C2	FPNAPSTDMSA	Omp19	HLA-B*54:01	CTL (MHC-I)	3
REC1-C3	ATDNVLLRLEY	Omp25	HLA-A*01:01	CTL (MHC-I)	1
REC1-C4	MVYGTGGLAY	Omp31	HLA-B*15:02	CTL (MHC-I)	13
REC1-C5	RASVGVSLMW	BamA	HLA-B*58:01	CTL (MHC-I)	1
REC1-T1	QAMYEAAQAAEGAGA	DnaK	HLA-DRB1*10:01	Th (MHC-II)	26
REC1-T2	KQLVLYDANGGTVAS	Omp19	HLA-DRB1*15:01	Th (MHC-II)	4
REC1-T3	TSTSAYAADAIVAQE	Omp25	HLA-DQA1*01:02/DQB1*06:09	Th (MHC-II)	10
REC1-T4	TGGYIGINAGYAGGK	Omp31	HLA-DRB1*10:01	Th (MHC-II)	17
REC1-T5	KTTFKGNYYQTLSQE	BamA	HLA-DPA1*01:03/DPB1*04:02	Th (MHC-II)	7

Note: SB Allele Count gives the number of strong-binding restricting alleles per epitope; the complete allele/heterodimer list is provided in Supplementary Table S3. For MHC-II, DQ and DP entries are functional α/β heterodimers predicted by NetMHCIIpan-4.1, whereas DR entries are DRB1 alleles.

**Table 7:** Predicted physicochemical parameters of REC1.

Physicochemical Parameters	REC1
Number of amino acids	357
Theoretical *pI*	5.64
Molecular weight (Da)	35,863.58
Total number of negatively charged residues (Asp + Glu)	38
Total number of positively charged residues (Arg + Lys)	32
Atomic composition	C_1590_H_2509_N_427_O_496_S_10_
Total number of atoms	5032
Estimated half-life	30 h (mammalian reticulocytes, in vitro) >20 h (yeast, in vivo) >10 h (*Escherichia coli*, in vivo)
Instability index (II)	25.25
Aliphatic index	79.89
GRAVY	−0.015

## Data Availability

The data is contained within the article or Supplementary Material.

## Supplementary Materials

The following supporting information can be downloaded at https://www.mdpi.com/article/10.3390/vaccines14100891/s1: Table S1: High-frequency HLA class I alleles in the Chinese population (*n* = 49); Table S2: High-frequency HLA class II alleles in the Chinese population (*n* = 71); Table S3: Complete list of strong-binding (SB) HLA restricting alleles/heterodimers for each epitope incorporated into REC1; Table S4: Conservation analysis of candidate proteins across major pathogenic *Brucella* species; Table S5: Predicted population coverage of the final REC1 epitope set in the China Oriental population (IEDB, with average hits and PC90); Table S6: Allele retention funnel and cumulative population carrier coverage of the Chinese HLA screening panels at the ≥3% carrier frequency cutoff; Table S7: Chinese DQA1-DQB1 cis-haplotype frequencies and Hardy-Weinberg-derived HLA-DQ population coverage (strict and relaxed); Table S8: Characteristics of the 12 shortlisted candidate immunoreactive epitopes, including predicted MHC-binding anchor and TCR-facing positions; Table S9: Epitope-level sequence conservation across eight major *Brucella* species; Table S10: Molecular docking interface metrics for REC1, its modules, and source antigens against innate receptors; Figure S1: Frequency-ranked cumulative carrier coverage curves of the Chinese HLA screening panels; Figure S2: AlphaFold 3 confidence for full-length REC1; Figure S3: Extended innate-sensor docking of the L7/L12 carrier: CD14, C1q globular head and DC-SIGN CRD; Figure S4: Individual source antigen docking controls against TLR2: DnaK, Omp19, Omp25, Omp31 and BamA.

## Abbreviations

The following abbreviations are used in this manuscript:

CD8^+^ cytotoxic T (Tc/CTL) cell	CTL-cell
HLA	human leukocyte antigen
MHC	Major Histocompatibility Complex
Allele Frequency Net Database	AFND
CD4^+^ helper T (Th) cell	Th-cell
Immune Epitope Database	IEDB
Spot-forming unit	SFU
Grand average of hydropathicity	GRAVY
Isoelectric point	pI
Plasmablast	PLB
Natural killer	NK
Dendritic cells	DCs
Macrophages	MAs
