# Supplementary material for: Development of REC1: A Reverse Vaccinology-Derived Multi-Epitope Vaccine Candidate Against Brucellosis
Source: Vaccines (Basel). 2026 Oct 12;14(10):891. doi: 10.3390/vaccines14100891 (PMC13709728; doi:10.3390/vaccines14100891)
Supplement: Supplementary file 1 [file vaccines-14-00891-s001.zip › Table S1.pdf]

vaccines

MDPI

Table S1. High-frequency HLA class I alleles in the Chinese population (*n* = 49).

No.	HLA Allele	Population Frequency (%)
1	HLA-A*11:01	52.3
2	HLA-C*01:02	40.5
3	HLA-C*07:02	33.8
4	HLA-B*15:02	33.0
5	HLA-A*24:02	31.8
6	HLA-B*46:01	30.0
7	HLA-B*40:01	28.2
8	HLA-C*03:04	27.5
9	HLA-A*02:07	25.6
10	HLA-A*02:01	20.3
11	HLA-C*08:01	15.7
12	HLA-A*33:03	14.8
13	HLA-C*06:02	14.2
14	HLA-C*03:03	13.7
15	HLA-B*58:01	12.3
16	HLA-C*03:02	12.2
17	HLA-B*13:01	11.4
18	HLA-C*04:01	10.5
19	HLA-B*51:01	10.3
20	HLA-B*15:01	9.7
21	HLA-B*13:02	8.8
22	HLA-C*14:02	8.4
23	HLA-A*30:01	8.1
24	HLA-A*02:06	7.5
25	HLA-A*02:03	7.1
26	HLA-B*55:02	6.4
27	HLA-C*15:02	6.2
28	HLA-B*54:01	6.2
29	HLA-A*24:07	5.8
30	HLA-A*31:01	5.7
31	HLA-A*26:01	5.3
32	HLA-B*35:01	5.1
33	HLA-B*40:06	5.0
34	HLA-C*12:02	4.9
35	HLA-B*56:01	4.9
36	HLA-B*15:25	4.9
37	HLA-A*11:02	4.8
38	HLA-A*01:01	4.6
39	HLA-B*38:02	4.3
40	HLA-C*02:02	4.2
41	HLA-B*52:01	4.0
42	HLA-B*39:01	4.0
43	HLA-A*68:01	3.9
44	HLA-B*40:02	3.6
45	HLA-B*44:03	3.5
46	HLA-A*03:01	3.5
47	HLA-B*48:01	3.4

48	HLA-B*07:02	3.2
49	HLA-B*15:11	3.0
