# Supplementary material for: Development of REC1: A Reverse Vaccinology-Derived Multi-Epitope Vaccine Candidate Against Brucellosis
Source: Vaccines (Basel). 2026 Oct 12;14(10):891. doi: 10.3390/vaccines14100891 (PMC13709728; doi:10.3390/vaccines14100891)
Supplement: Supplementary file 1 [file vaccines-14-00891-s001.zip › Table S10.pdf]

**Table S10.** Molecular docking interface metrics for REC1, its modules, and source antigens against innate receptors.

| Group | Cluster size | HADDOCK score | BSA (Å²) | H-bonds (atom pairs) | Salt bridges | Hydrophobic | π | Low-conf. fraction | Pocket hit |
|---|---|---|---|---|---|---|---|---|---|
| L7/L12-TLR2 | 112 | -98.3 | 1025 | 17 | 1 | 6 | 2 | 0.105 | Yes |
| L7/L12-TLR4/MD-2 | 110 | -250.6 | 1324 | 23 | 5 | 3 | 1 | 0.29 | Yes |
| REC1 full-TLR2 | 10 | -100.1 | 1189 | 26 | 0 | 11 | 2 | 0.50 | Yes |
| REC1 full-TLR4/MD-2 | 5 | -265.4 | 1654 | 22 | 3 | 23 | 1 | 0.48 | Yes |
| CTL array-TLR2 | 6 | -75.9 | 991 | 16 | 0 | 4 | 1 | 0.56 | Yes |
| Th array-TLR4/MD-2 | 7 | -265.2 | 1378 | 21 | 2 | 6 | 0 | 1.00 | Yes |
| DnaK-TLR2 | 4 | -69.6 | 778 | 16 | 1 | 5 | 1 | 0.11 | Yes |
| Omp19-TLR2 | 8 | -97.1 | 1089 | 18 | 1 | 13 | 1 | 0.32 | Yes |
| Omp25-TLR2 | 4 | -100.3 | 1189 | 15 | 1 | 22 | 1 | 0.00 | Yes |
| Omp31-TLR2 | 8 | -91.0 | 953 | 8 | 0 | 9 | 0 | 0.00 | Yes |
| BamA-TLR2 | 4 | -132.0 | 1460 | 10 | 1 | 16 | 7 | 0.00 | Yes |
| L7/L12-CD14 | 98 | -101.5 | 798 | 18 | 4 | 9 | 1 | 0.00 | Yes |
| L7/L12-C1q | 34 | -453.6 | 536 | 9 | 3 | 0 | 0 | 0.00 | Yes |
| L7/L12-DC-SIGN | 79 | -120.4 | 712 | 18 | 4 | 2 | 1 | 0.00 | Yes |
| REC1 full-CD14 | 6 | -105.1 | 913 | 16 | 2 | 1 | 3 | 1.00 | Yes |
| REC1 full-C1q | 34 | -463.4 | 479 | 2 | 0 | 4 | 0 | 0.89 | No |
| REC1 full-DC-SIGN | 6 | -120.6 | 602 | 20 | 3 | 0 | 1 | 1.00 | Yes |
