# Supplementary material for: Development of REC1: A Reverse Vaccinology-Derived Multi-Epitope Vaccine Candidate Against Brucellosis
Source: Vaccines (Basel). 2026 Oct 12;14(10):891. doi: 10.3390/vaccines14100891 (PMC13709728; doi:10.3390/vaccines14100891)
Supplement: Supplementary file 1 [file vaccines-14-00891-s001.zip › Table S2.pdf]

Table S2. High-frequency HLA class II alleles in the Chinese population (*n* = 71).

No.	HLA Allele	Population Frequency (%)
1	HLA-DQB1*02:01	53.5
2	HLA-DPB1*04:01	50.9
3	HLA-DQA1*03	48.5
4	HLA-DQA1*05:01	48.1
5	HLA-DQB1*03:01	42.9
6	HLA-DQA1*02:01	39.4
7	HLA-DQB1*03:03	38.0
8	HLA-DRB1*07:01	37.0
9	HLA-DRB1*09:01	35.8
10	HLA-DQA1*01:02	34.3
11	HLA-DPB1*02:01	33.3
12	HLA-DQA1*01:01	32.5
13	HLA-DQA1*01:03	30.0
14	HLA-DRB1*13:01	27.3
15	HLA-DRB1*15:01	26.5
16	HLA-DRB1*03:01	26.3
17	HLA-DQB1*06:01	24.0
18	HLA-DQB1*05:01	23.1
19	HLA-DQB1*06:02	21.4
20	HLA-DQB1*03:02	21.1
21	HLA-DRB1*13:02	19.6
22	HLA-DRB1*11:01	18.2
23	HLA-DRB1*04:03	17.7
24	HLA-DRB1*14:01	17.7
25	HLA-DRB1*15:02	17.7
26	HLA-DPB1*04:02	17.5
27	HLA-DRB1*12:01	17.5
28	HLA-DRB1*12:02	16.8
29	HLA-DRB1*08:03	15.9
30	HLA-DQB1*05:02	15.7
31	HLA-DQB1*06:04	15.5
32	HLA-DRB1*15:04	14.4
33	HLA-DPB1*05:01	14.0
34	HLA-DPB1*14:01	14.0
35	HLA-DRB1*11:04	13.0
36	HLA-DQA1*06:01	12.9
37	HLA-DQB1*02:02	12.9
38	HLA-DRB1*10:01	12.1
39	HLA-DRB1*14:03	11.8
40	HLA-DQB1*05:03	11.4
41	HLA-DQB1*06:03	11.3
42	HLA-DQB1*04:01	10.7
43	HLA-DRB1*16:02	10.7
44	HLA-DPB1*17:01	10.5
45	HLA-DRB1*11:03	10.5
46	HLA-DRB1*04:05	9.9
47	HLA-DPB1*02:02	9.7

48	HLA-DQB1*04:02	9.6
49	HLA-DRB1*04:01	9.1
50	HLA-DRB1*08:02	9.1
51	HLA-DPB1*03:01	8.8
52	HLA-DPB1*13:01	8.8
53	HLA-DRB1*04:06	8.7
54	HLA-DQB1*06:09	8.6
55	HLA-DRB1*14:05	7.5
56	HLA-DRB1*12:03:02	7.4
57	HLA-DRB1*14:54	7.1
58	HLA-DRB1*08:01	6.1
59	HLA-DRB1*04:07	5.9
60	HLA-DQA1*04:01	5.8
61	HLA-DRB1*01:01:01	5.8
62	HLA-DRB1*04:04	5.3
63	HLA-DRB1*04:08	5.3
64	HLA-DRB1*01:01	4.9
65	HLA-DRB1*13:03	4.4
66	HLA-DRB1*14:04	4.4
67	HLA-DPB1*01:01	3.5
68	HLA-DPB1*09:01	3.5
69	HLA-DRB1*01:02	3.5
70	HLA-DRB1*04:02	3.5
71	HLA-DRB1*11:06:01	3.1
