# Supplementary material for: Development of REC1: A Reverse Vaccinology-Derived Multi-Epitope Vaccine Candidate Against Brucellosis
Source: Vaccines (Basel). 2026 Oct 12;14(10):891. doi: 10.3390/vaccines14100891 (PMC13709728; doi:10.3390/vaccines14100891)
Supplement: Supplementary file 1 [file vaccines-14-00891-s001.zip › Table S3.pdf]

vaccines

MDPI

Table S3. Complete list of strong binding (SB) HLA restricting alleles/heterodimers for each epitope incor-porated into REC1.

Epitope ID	Sequence	Type	All strong-binding restricting HLA alleles / het-erodimers
REC1-C1	LQKMKETAESY	CTL (MHC-I)	HLA-B*15:01; HLA-B*15:25; HLA-B*15:02
REC1-C2	FPNAPSTDMSA	CTL (MHC-I)	HLA-B*54:01; HLA-B*55:02; HLA-B*56:01
REC1-C3	ATDNVLLRLEY	CTL (MHC-I)	HLA-A*01:01
REC1-C4	MVYGTGGLAY	CTL (MHC-I)	HLA-B*15:02; HLA-B*15:25; HLA-B*15:01; HLA-A*26:01; HLA-B*46:01; HLA-B*15:11; HLA-B*35:01; HLA-C*03:02; HLA-C*12:02; HLA-A*03:01; HLA-C*02:02; HLA-C*14:02; HLA-A*01:01
REC1-C5	RASVGVSLMW	CTL (MHC-I)	HLA-B*58:01
REC1-T1	QAMYE-AAQAAEGAGA	Th (MHC-II)	HLA-DRB1*10:01; HLA-DRB1*09:01; HLA-DQA1*03:01/DQB1*03:03; HLA-DQA1*05:01/DQB1*03:01; HLA-DRB1*01:01; HLA-DRB1*04:07; HLA-DQA1*04:01/DQB1*04:02; HLA-DQA1*06:01/DQB1*04:01; HLA-DRB1*04:01; HLA-DRB1*04:08; HLA-DRB1*16:02; HLA-DRB1*08:02; HLA-DQA1*01:02/DQB1*06:01; HLA-DQA1*01:01/DQB1*05:03; HLA-DQA1*01:02/DQB1*06:02; HLA-DQA1*03:01/DQB1*03:02; HLA-DQA1*01:02/DQB1*06:09; HLA-DRB1*14:03; HLA-DQA1*01:02/DQB1*05:02; HLA-DRB1*11:01; HLA-DRB1*07:01; HLA-DQA1*01:03/DQB1*06:03; HLA-DRB1*04:05; HLA-DRB1*08:03; HLA-DQA1*02:01/DQB1*02:01; HLA-DQA1*02:01/DQB1*02:02
REC1-T2	KQLVLYDANGGT-VAS	Th (MHC-II)	HLA-DRB1*15:01; HLA-DRB1*15:04; HLA-DRB1*15:02; HLA-DRB1*04:02
REC1-T3	TSTSAYAADAI-VAQE	Th (MHC-II)	HLA-DQA1*01:02/DQB1*06:09; HLA-DQA1*01:02/DQB1*06:04; HLA-DQA1*01:03/DQB1*06:03; HLA-DQA1*03:01/DQB1*03:03; HLA-DQA1*01:02/DQB1*06:01; HLA-DQA1*05:01/DQB1*03:01; HLA-DRB1*04:01; HLA-DQA1*01:02/DQB1*06:02; HLA-DQA1*03:01/DQB1*03:02; HLA-DQA1*01:01/DQB1*05:03
REC1-T4	TGGYIGI-NAGYAGGK	Th (MHC-II)	HLA-DRB1*10:01; HLA-DRB1*08:02; HLA-DQA1*04:01/DQB1*04:02; HLA-DRB1*16:02; HLA-DRB1*01:01; HLA-

REC1-T5	KTT-FKGNYYQTLSQE	Th (MHC-II)	DQA1*06:01/DQB1*04:01; HLA-DRB1*04:08; HLA-DRB1*04:07; HLA-DQA1*03:01/DQB1*03:03; HLA-DRB1*11:01; HLA-DRB1*14:03; HLA-DRB1*04:01; HLA-DRB1*08:03; HLA-DRB1*08:01; HLA-DRB1*15:02; HLA-DRB1*04:05; HLA-DRB1*09:01 HLA-DPA1*01:03/DPB1*04:02; HLA-DPA1*01:03/DPB1*02:01; HLA-DPA1*01:03/DPB1*02:02; HLA-DPA1*01:03/DPB1*04:01; HLA-DPA1*01:03/DPB1*01:01; HLA-DPA1*01:03/DPB1*05:01; HLA-DPA1*01:03/DPB1*13:01
