# Supplementary material for: Development of REC1: A Reverse Vaccinology-Derived Multi-Epitope Vaccine Candidate Against Brucellosis
Source: Vaccines (Basel). 2026 Oct 12;14(10):891. doi: 10.3390/vaccines14100891 (PMC13709728; doi:10.3390/vaccines14100891)
Supplement: Supplementary file 1 [file vaccines-14-00891-s001.zip › Table S4.pdf]

vaccines

MDPI

Table S4. Conservation analysis of candidate proteins across major pathogenic *Brucella* species.

Source antigen Identities	*B. melitensis 16M*	*B. abortus 2308*	*B. suis 1330*	*B. ovis*	*B. microti*	*B. canis*	*B. pinnipedialis*	*B. ceti*
GroEL	100%	99.8%	99.6%	99.6%	99.6%	99.6%	99.6%	99.5%
DnaK	100%	99.8%	99.7%	99.8%	99.8%	99.7%	99.8%	99.8%
Omp19	100%	100%	100%	99.4%	100%	100%	100%	100%
Omp31	100%	-	100%	97.1%	100%	99.6%	100%	100%
Omp25	100%	100%	100%	100%	100%	100%	100%	100%
BamA	100%	99.6%	99.6%	99.7%	99.7%	99.7%	99.7%	99.6%
