# Supplementary material for: Development of REC1: A Reverse Vaccinology-Derived Multi-Epitope Vaccine Candidate Against Brucellosis
Source: Vaccines (Basel). 2026 Oct 12;14(10):891. doi: 10.3390/vaccines14100891 (PMC13709728; doi:10.3390/vaccines14100891)
Supplement: Supplementary file 1 [file vaccines-14-00891-s001.zip › Table S5.pdf]

Table S5. Predicted population coverage of the final REC1 epitope set in the China Oriental population (IEDB, with average hits and PC90).

Analysis group	Restriction set used	Coverage (%)	Average hits per individual	PC90
MHC-I only	5 CTL epitopes, all class-1 strong-binding alleles	71.39	1.37	0.35
MHC-II (DR-only)	Four DR-restricted Th epitopes (DRB1 alleles)	69.15	1.50	0.32
Combined I + DR (conservative main result)	5 CTL + four DR-restricted Th epitopes	91.17	2.86	1.09
MHC-II (DR + DP approximate)	Four DR + one DP-restricted Th epitope (DPB1-level approximation, fixed DPA1*01:03)	96.10	2.69	1.32
Combined I + DR +DP (extended upper bound)	5 CTL + five Th epitopes (DR + DP approximate)	98.88	4.05	2.17

Table note: Calculations were performed using IEDB Population Coverage tool for China-Oriental population. PC90 denotes the minimal number of epitopes-HLA matches achieved by at least 90% of the population. HLA-DQ coverage is reported separately and is not integrated in this IEDB-derived table.
