# Supplementary material for: Development of REC1: A Reverse Vaccinology-Derived Multi-Epitope Vaccine Candidate Against Brucellosis
Source: Vaccines (Basel). 2026 Oct 12;14(10):891. doi: 10.3390/vaccines14100891 (PMC13709728; doi:10.3390/vaccines14100891)
Supplement: Supplementary file 1 [file vaccines-14-00891-s001.zip › Table S6.pdf]

Table S6. Allele retention funnel and cumulative population carrier coverage of the Chinese HLA screening panels at the ≥3% carrier frequency cutoff.

HLA class	Locus/group	Raw AFND records	Non-zero carrier-frequency records	Distinct non-zero labels	Included panel labels (≥3%)	Excluded labels (< 3%)	All positive four-digit alleles	Panel cumulative coverage (≥ 3%)	Full-tall coverage (%)	Long-tall gain (pp)
I	HLA class I (A + B + C)	5647	428	179	49	130	178	99.90	99.95	0.05
I	HLA-A	1631	121	47	15	32	46	90.80	91.67	0.87
I	HLA-B	3012	218	88	22	66	88	89.70	92.54	2.83
I	HLA-C	1004	89	44	12	32	44	89.04	91.20	2.16
II	HLA class II (overall)	3010	561	124	71	53	123	-	-	-
II	HLA-DRB1	1762	335	66	37	29	66	99.51	99.57	0.07
II	HLA-DQA1	132	49	8	8	0	7	91.99	91.99	0
II	HLA-DQB1	500	142	27	15	12	27	97.85	97.94	0.09
II	HLA-DPA1	15	0	0	0	0	-	-	-	-
II	HLA-DPB1	601	35	23	11	12	23	87.49	88.94	1.45
