# Supplementary material for: Development of REC1: A Reverse Vaccinology-Derived Multi-Epitope Vaccine Candidate Against Brucellosis
Source: Vaccines (Basel). 2026 Oct 12;14(10):891. doi: 10.3390/vaccines14100891 (PMC13709728; doi:10.3390/vaccines14100891)
Supplement: Supplementary file 1 [file vaccines-14-00891-s001.zip › Table S7.pdf]

Table S7. Chinese DQA1-DQB1 cis haplotype frequencies and Hardy-Weinberg-derived HLA-DQ population coverage (strict and relaxed).

Haplotype identifier	Han frequency (%)	Hui frequency (%)	Kazakh frequency (%)	Uyghur frequency (%)
DQA1*03:01-DQB1*03:03	0	0	0	0
DQA1*05:01-DQB1*03:01	10.00	6.25	10.58	10.56
DQA1*04:01-DQB1*04:02	0	2.50	1.92	1.41
DQA1*06:01-DQB1*04:01	0	0	0	0
DQA1*01:02-DQB1*06:01	3.57	0	0	0
DQA1*01:01-DQB1*05:03	5.00	2.50	0.96	4.93
DQA1*01:02-DQB1*06:02	7.86	6.25	4.81	3.52
DQA1*03:01-DQB1*03:02	0	0	0	0
DQA1*01:02-DQB1*06:09	4.29	3.75	2.88	1.41
DQA1*01:02-DQB1*05:02	2.86	0	0.96	2.11
DQA1*01:03-DQB1*06:03	2.86	5.00	2.88	4.93
DQA1*02:01-DQB1*02:01	6.43	8.75	10.58	18.31
DQA1*02:01-DQB1*02:02	0	0	0	0
DQA1*01:02-DQB1*06:04	0.71	0	0	7.04
DQA1*03→03:01-DQB1*03:03 (low-resolution, relaxed)	7.14	11.25	4.81	2.11
DQA1*03→03:01-DQB1*03:02 (low-resolution, relaxed)	5.71	6.25	5.77	4.93
Sum of target haplotypes s (strict, %)	43.58	35.00	35.57	54.22
Phenotypic coverage $C = 2s - s^2$ (strict, %)	68.17	57.75	58.49	79.04
Sum of target haplotypes s (relaxed, %)	56.43	52.50	46.15	61.26
Phenotypic coverage $C = 2s - s^2$ (relaxed, %)	81.02	77.44	71.00	84.99
