# Supplementary material for: Development of REC1: A Reverse Vaccinology-Derived Multi-Epitope Vaccine Candidate Against Brucellosis
Source: Vaccines (Basel). 2026 Oct 12;14(10):891. doi: 10.3390/vaccines14100891 (PMC13709728; doi:10.3390/vaccines14100891)
Supplement: Supplementary file 1 [file vaccines-14-00891-s001.zip › Table S8.pdf]

vaccines

MDPI

Table S8. Characteristics of the 12 shortlisted candidate immunoreactive epitopes, including predicted MHC-binding anchor and TCR-facing positions.

Epitope ID	Epitope sequence	Length	Position in antigen (aa)	NetMHCpan EL icore	NetMHCpan/IIpan EL core	Predicted MHC anchors	Predicted TCR-facing (+ bulge)
C1	LQKMKETAESY	11	120-130	LQKMKETAESY	LQKETAESY	p2=Q pΩ=Y	P3-P8=KETAES + bulge MK
C2	FPNAPSTDMSA	11	60-70	FPNAPSTDMSA	FPNAPSMSA	p2=P pΩ=A	P3-P8=NAPSMS + bulge TD
C3	ATDNVLLRLEY	11	186-196	ATDNVLLRLEY	ATDNVRLEY	p3=D pΩ=Y	P2、P4-P8=TNVRLE + bulge LL
C4	MVYGTGGLAY	10	149-158	MVYGTGGLAY	MVYGGGLAY	p2=V pΩ=Y	P3-P8=YGGGLA + bulge T
C5	RASVGVSLMW	10	738-747	RASVGVSLMW	RSVGVSLMW	p2=S pΩ=W	P3-P8=VGVSLM + bulge A
P9	SSNAVLDPSTNEY	13	254-266	SSNAVLDPSTNEY	SSDPSTNEY	p3=D pΩ=Y	P2、P4–P8=SPSTNE + bulge NAVL
P12	YSVDQGPRAY	10	352-361	YSVDQGPRAY	YSDQGPRAY	p3=D pΩ=Y	P2、P4-P8=SQGPRA + bulge V
T1	QAMYEAAQAAEGAGA	15	595-609		YEAAQAAEG	P1=Y, P4=A, P6=A, P9=G	P2/P3/P5/P7/P8=EAQAE
T2	KQLVLYDANGGTVAS	15	140-154		LVLYDANGG	P1=L, P4=Y, P6=A, P9=G	P2/P3/P5/P7/P8=VLDNG
T3	TSTSAYAADAIVAQE	15	16-30		SAYAADAIV	P1=S, P4=A, P6=D, P9=V	P2/P3/P5/P7/P8=AYAAI
T4	TGGYIGINAGYAGGK	15	41-55		YIGINAGYA	P1=Y, P4=I, P6=A, P9=A	P2/P3/P5/P7/P8=IGNGY
T5	KTTFKGNYYQTLSQE	15	615-629		FKGNYYQTL	P1=F, P4=N, P6=Y, P9=L	P2/P3/P5/P7/P8=KGYQT
