# Supplementary material for: Development of REC1: A Reverse Vaccinology-Derived Multi-Epitope Vaccine Candidate Against Brucellosis
Source: Vaccines (Basel). 2026 Oct 12;14(10):891. doi: 10.3390/vaccines14100891 (PMC13709728; doi:10.3390/vaccines14100891)
Supplement: Supplementary file 1 [file vaccines-14-00891-s001.zip › Table S9.pdf]

vaccines

MDPI

Table S9. Epitope-level sequence conservation across eight major *Brucella* species.

Epitope	*B. melitensis 16M*	*B. abortus 2308*	*B. suis 1330*	*B. ovis*	*B. microti*	*B. canis*	*B. pinnipedialis*	*B. ceti*
C1-LQKMKETAESY-DnaK	100%	100%	100%	100%	100%	100%	100%	100%
C2-FPNAPSTDMSA-Omp19	100%	100%	100%	100%	100%	100%	100%	100%
C3-ATDNVLLRLEY-Omp25	100%	100%	100%	100%	100%	100%	100%	100%
C4-MVYGTGGLAY-Omp31	100%	absent	100%	100%	100%	100%	100%	100%
C5-RASVGVSLMW-BamA	100%	100%	100%	100%	100%	100%	100%	100%
P9-SSNAVLDPSTNEY-BamA	100%	100%	100%	100%	100%	100%	100%	100%
P12-YSVDQGPRAY-BamA	100%	100%	100%	100%	100%	100%	100%	100%
T1-QAMYEAAQAAEGAGA-DnaK	100%	100%	100%	100%	100%	100%	100%	100%
T2-KQLVLYDANGGTVAS-Omp19	100%	100%	100%	100%	100%	100%	100%	100%
T3-TSTSAYAADAIVAQE-Omp25	100%	100%	100%	100%	100%	100%	100%	100%
T4-TGGYIGINAGYAGGK-Omp31	100%	absent	100%	100%	100%	100%	100%	100%
T5-KTTFKGNYYQTLSQE-BamA	100%	100%	100%	100%	100%	100%	100%	100%
